# Regulating Companion Dog Welfare: A Comparative Study of Legal Frameworks in Western Countries

**DOI:** 10.3390/ani11061660

**Published:** 2021-06-02

**Authors:** Søren Stig Andersen, Iben Meyer, Björn Forkman, Søren Saxmose Nielsen, Peter Sandøe

**Affiliations:** 1 Department of Food and Resource Economics, University of Copenhagen, DK-1958 Frederiksberg C, Denmark; 2 Department of Veterinary and Animal Sciences, University of Copenhagen, DK-1870 Frederiksberg C, Denmark; iben@dyreadfaerdskonsulenten.dk (I.M.); bjf@sund.ku.dk (B.F.); saxmose@sund.ku.dk (S.S.N.)

**Keywords:** dogs, companion animals, animal welfare, regulation, breeding, sales, surgery, killing, legislation, legal comparison

## Abstract

**Simple Summary:**

Animal welfare legislation is an important tool defining acceptable minimum requirements for the breeding and guardianship of animals. With farm and laboratory animals, there has been a focus on the role of legislation, and considerable efforts have been made to compare and harmonise animal welfare legislation across countries. The role of legislation in maintaining the welfare of companion animals has received less attention. We rectify this by examining and comparing legislation on the welfare of companion dogs in eleven Western jurisdictions. The welfare issues we look at include breeding, surgical interventions, day-to-day handling, and killing. We demonstrate that there is significant variation across the jurisdictions in the way the legislation is developing. Thus, some countries have implemented regulations setting out how private owners should take care of their dogs (e.g., by taking them for walks), while others have not. Most of the countries studied regulate sales of dogs (e.g., requiring a minimum age when sold) and ban surgical interventions such as ear cropping and debarking. The main exception is the USA, where despite some differences at state and municipality level, the regulation is in general minimal.

**Abstract:**

There appear to be growing concerns among experts, NGOs, and members of the public about the welfare of companion dogs. With farm and laboratory animals, legislative initiatives have long been considered valuable tools in the management of welfare whereas the use of legislation to protect companion animal welfare has received less attention. We aim to rectify this by comparing legislation with an impact on the welfare of companion dogs in eleven Western jurisdictions. The comparison also provides a basis for further consideration of regulatory initiatives. We identify the rules applying in the jurisdictions and classify them in accordance with the following categories: breeding of dogs with risks to the health of the offspring, reproductive limitations, sales, surgical interventions, day-to-day handling, and killing. We demonstrate that, overall, there is significant variation across the jurisdictions. However, the degree of variation depends on the specific category. Whereas most countries, with the USA being a notable exception, regulate sales of dogs and ban surgical interventions, there is considerable variation in the regulation of day-to-day handling and the killing of dogs. Furthermore, different jurisdictions employ different regulatory tools to ensure the desired level of welfare for companion dogs. Overall, there appears to be real potential for dialogue and mutual inspiration.

## 1. Introduction

For decades, many Western countries have concentrated their animal welfare policies and regulatory initiatives on production animals and laboratory animals. In Europe, this focus is reflected in, and has been sustained by, the adoption of legal instruments regulating various aspects of welfare in production animals under the EU Common Agricultural Policy and within the framework of the Council of Europe [1]. Worldwide, there has also been increasing coordination and convergence in the use of animals for research purposes, which now very often requires an assessment by an ethics committee and must satisfy ethical criteria [2]. By contrast, regulatory initiatives designed to protect the welfare of companion animals have been slower to develop, both in terms of scope (i.e., the range of welfare aspects covered) and specificity (i.e., the degree of detail of the requirements). Indeed, in many jurisdictions, there has been little legislation over and above the general anti-cruelty and animal welfare statutes [3] (p. 256).

It appears, however, that the welfare of companion animals such as companion dogs is beginning to receive more attention from policy makers and legislators, as well as academics [3]. In this article, we examine the current state of the legislation applying to privately owned dogs, comparing the different legislative strategies that various Western jurisdictions have employed. To our knowledge, this kind of study has not previously been carried out.

The welfare of companion dogs is affected by a number of factors. We focus on the following, where the animal’s welfare is directly impacted rather than being a secondary effect: breeding of dogs with potentially adverse welfare characteristics, reproductive limits (on minimum age for mating and number of litters), sales (point of sale, minimum age limit for puppies on sale, and mandatory care instructions), surgical interventions (neutering, tail docking, ear cropping, and debarking), day-to-day handling by owner (design of indoor and outdoor environment, dog walking, duration of time spent alone, prolonged restraint, use of electric collars), and killing (including euthanasia).

We examine eight European jurisdictions and three Western but non-European jurisdictions (New South Wales in Australia, New Zealand, and the USA). By identifying and classifying the rules in these Western jurisdictions, we are able to develop a comparative analysis illuminating differences and similarities in the legislative options and traditions characterising the respective countries [4].

Our investigation reveals differences between the jurisdictions, with variation in both the scope and the specificity of the regulations. Furthermore, the comparative analysis we present helps to reveal some conceptual and ideological frameworks that underlie these differences [5]. A central question is whether the regulatory differences that we catalogue arise from variation in the legal traditions of the jurisdictions or instead reflect distinctive attitudes to dogs, or animals in general, in the relevant jurisdictions.

Finally, we look at the different legislative mechanisms (parliamentary laws, binding administrative regulations, non-binding codes of conduct, etc.) employed in different jurisdictions.

It should be stressed from the outset that even though our study is motivated by an interest in the welfare of companion dogs, our focus is on the content of the legislation set up to protect dog welfare, whereas we are not here trying to assess the extent to which the purpose of the legislation is actually reached through implementation and enforcement.

## 2. Materials and Methods

The article is based on an examination of the animal welfare legislation applying to companion dogs in the following jurisdictions: Austria, Denmark, England, Germany, Italy, the Netherlands, New South Wales, New Zealand, Norway, Sweden, and the USA. Where Germany and Austria were concerned, we investigated both the federal legislation and the laws in three federal states that exhibited peculiarities of special interest vis-à-vis the legislation on state level. In the USA and Australia, much of the legislation, as in Germany and Austria, has been developed at state level, and, in the case of the USA, even at times at a lower administrative level, e.g., county or town level. Therefore, for the USA, we provide a summary covering states and counties that is based on interviews with two American scholars in the field and relevant articles on the Michigan State University animal law website [6]. Where possible, we have indicated where, and in what way, the legislation may vary between states and counties. However, a general caveat needs to be entered here: the brief summary contained in this article is not intended to capture all of the details and nuances of the legislative geography. For Australia, we selected a single state, New South Wales. We also decided to refer to the law of England, since although some of the legislation we refer to applies throughout the UK, the legislative framework applying in Northern Ireland, Scotland, and Wales differs in some cases from that applying in England.

In each jurisdiction, we have identified and classified the dog-related legislation with reference to the following welfare issues:

- Breeding of dogs with potentially adverse welfare characteristics;
- Reproductive limits (on minimum age for mating and number of litters);
- Sales (point of sale, minimum age for puppies on sale, and mandatory care instructions);
- Surgical interventions (neutering, tail docking, ear cropping, and debarking);
- Day-to-day handling by owners (design of indoor and outdoor environment, dog walking, duration of time spent alone, use of collars);
- Killing (incl. euthanasia).

Information on the legislation in individual jurisdictions was derived partly from legal texts, and partly from information on the websites of relevant authorities. In some cases, we obtained the information from other texts and websites originating from, for example, lawyers specialising in companion animal law. In some jurisdictions, information was also obtained from guidelines prepared by the authorities or, in the case of the USA, as mentioned, through interviews with experts.

### 2.1. Scope of the Investigation

The study concerns privately owned companion dogs, as opposed to dogs kept and used for other purposes, such as research, policing, or military use. Clearly, dogs can be working dogs—e.g., guide dogs, farm dogs, hunting dogs, police dogs, etc.—while at the same time being treated as a companion animal, at least some of the time. We did not examine any special issues created by companion dogs also serving as working dogs.

Another aspect of the concept of a ‘companion dog’ requiring clarification arises from the distinction between commercial and non-commercial ownership. Although both situations encompass companion dogs, dogs kept for breeding and/or sale are often covered by a higher level of regulation (both in terms of scope and specificity) than those kept for private use. Some countries clearly define commercial versus non-commercial ownership, while the distinction is more fluid in other countries. Our study focuses primarily on non-commercial situations, where an individual or a family owns one or a few dogs. However, since the purchase of a companion dog from a (professional or hobby-based) breeder involves elements of commercialism, in some cases, we also consider parts of the legislation applying to commercial situations.

A distinction also needs to be made between rules concerning the welfare of dogs and rules concerning other dog-related issues, where the latter typically relate to human health and safety. Although rules of the second kind here may well have an indirect effect on dog welfare, we confine our attention to legislation that more directly concerns dog welfare. We do not examine, for example, rules on animal imports and on ‘dangerous dogs’. However, the boundary between welfare-related regulation and other regulation is obviously a little fuzzy. It can be argued, for instance, that the purpose of muzzling is not only to protect humans and other dogs but also to improve dog welfare, as it enables owners to take their dogs for walks in public spaces. Rules concerning the use of a leash can be even harder to categorise. An obligation to use a leash may be primarily about the safety of humans and other animals, while rules relating to the type and length of the leash may primarily be about the dog’s welfare. Due to the rather indirect way in which they concern dog welfare, we have excluded both rules on muzzling and the use of leash from our investigation. Legislation on the vaccination of dogs is another area of regulation left out from our investigation, since it is not unambiguously related to the welfare of individual dogs.

Decisions about which issues to include in the investigation were furthermore based on their connection with other issues that are examined. We also prioritised welfare issues that are regulated in at least some of the jurisdictions under survey. A number of welfare issues were ignored, because they are addressed solely by guidelines, or other types of soft law, across the selected jurisdictions, such as recommendations concerning the care of older dogs or on treatment of various diseases. As mentioned above, we also excluded issues that are relevant predominantly in commercial situations, such as rules on insemination and on the training requirements for staff who engage with dogs in, for example, kennels and pet shops.

It should be emphasized that our comparison only concerns the wording of statute law and other relevant legislation. Our material does not enable us to address important issues such as enforcement and the actual effects of the legislation with regard to the welfare of dogs covered by the legislation in question.

### 2.2. Legal Characteristics of the Jurisdictions under Consideration

Traditionally, comparative law operates on the basis of a fundamental differentiation between civil law and common law jurisdictions. Whereas civil law is normally associated with statutory law and its associated codifications (e.g., legislation passed in a parliament and subsequently implemented in specific areas according to the purpose of the law), common law is constituted by case law, where a body of unwritten laws are implemented through previous rulings of the courts, known as precedents, particularly in cases where no written rules exist. In recent comparative legal studies, the significance and clarity of this distinction has been questioned. Indeed, Mark Van Hoecke and Mark Warrington went so far as to state, just over two decades ago, that the civil versus common law division was collapsing [5] (pp. 498–502).

Accordingly, the present study examines other differentiations of the legal terrain that are at least as important from an analytical point of view. Some countries, for instance, have centralised dog welfare regulation, whereas the regulation may vary between different administrative regions in other countries, especially of course in those with a more federal structure, such as Australia, Austria, Germany, and the USA.

Another distinction is between binding legal norms such as parliamentary laws and administrative regulations in the shape of ministerial orders or guidelines with legally binding standards, on the one hand, and non-binding guidelines, codes of conduct, and so on, typically containing more detailed recommendations for owners and persons in charge of dogs, on the other. In New South Wales and New Zealand, the authorities have issued ‘Codes of Practice’ and ‘Codes of Welfare’ that contain both legally binding standards and guidance (or recommended best practice). Likewise, Swedish regulation contains general guidelines. In Norway, the Norwegian Food Safety Authority has issued guidelines that assist its inspection staff in interpreting the provisions of the law. The English Code of Practice for the welfare of dogs serves a similar purpose.

The legal effect of the standards set out in the New South Wales and New Zealand codes is very similar to that of statutory provisions inasmuch as a contravention of these norms corresponds to an offence with regard to the underlying statutory provision. The guidelines, on the other hand, can be construed as soft law, because a contravention of a recommended norm usually does not in itself lead to a conviction. However, although the guidelines are not in themselves binding, they are likely to be very influential in the administrative and judicial application of the legislation. In the overview presented in Table 1 below (in Section 3), the different norms are assessed according to their intended legal effect, and not whether they appear in statutory law, a ministerial order, or in some guidelines or a code of conduct.

It also should be noted that in Europe, regional and intra-national legal instruments are used to regulate various dog-related issues across jurisdictions. Most prominently, the 1987 Council of Europe Convention for the Protection of Pet Animals, which has been ratified by most European countries (though not by the Netherlands, the UK, and some other countries not included in the present examination), imposes minimum requirements while leaving individual countries to decide on how to enforce these.

Furthermore, in 2007 the Lisbon Treaty established a principle according to which EU policies must have full regard for the welfare requirements of animals, while respecting the legislative or administrative provisions and customs of the member states (cf. Article 13 of the Treaty on the Functioning of the European Union). In regards to dog-related legislation, the EU has issued rules on cross-border trade [7] as well as movement [8] and transportation [9], but no rules that address welfare issues within the individual member states. Thus, even within the EU, welfare legislation applying to companion animals is almost entirely a national matter.

### 2.3. System of Indicators Enabling an Overall Comparison of the Countries

In comparing the different jurisdictions, we employ the following scale representing the degree of regulation of the various welfare issues:

- No regulation: 0;
- Low degree of regulation: 2;
- Medium degree of regulation: 4;
- High degree of regulation: 6.

Given the heterogenous nature of the welfare issues with which we are concerned, the indicative number is determined in any given case by an overall assessment of the various aspects of the issue in question rather than by applying a uniform formula.

## 3. Results

All of the jurisdictions we examined had general legislation prohibiting the ill-treatment of animals such as dogs. Invariably, this kind of legislation has implications for the welfare areas covered here. The following findings, however, relate solely to regulation concerning the particular welfare-related issue at hand.

Detailed information also covering some aspects not addressed in this paper, including links to the relevant legislation and other instruments, is available online at File S1.

### 3.1. Breeding of Dogs with Welfare-Threatening Characteristics (Health of Offspring)

Welfare problems can arise for large numbers of dogs in breeds with extreme phenotypes (e.g., brachycephalic breeds) or a high incidence of hereditary disease [3] (pp. 107–109). Denmark excepted, all of the selected European jurisdictions, as well as New South Wales and New Zealand, have rules which, in one way or another, outlaw the breeding of dogs with traits, defects, or serious abnormalities that can be expected to cause suffering either to the animal itself or to its offspring. In Austria and Norway, these rules concern human-bred animals in general. In the Netherlands, individual animals in the brachycephalic breeds must meet certain criteria if they are to be used in breeding. The criteria are specified in an expert opinion commissioned by the authorities. Likewise, a German expert opinion specifies defects disallowing dogs from being used for breeding and lists the breeds that experience problems with these defects.

Most legislation targets hereditary diseases in the offspring. In New South Wales, the bitch and male dog are required to be physically and mentally healthy when mating. Similar norms are part of the recommended practise in New Zealand. In Denmark, the Animal Welfare Act contains a delegation provision empowering the relevant government minister to issue rules on the breeding of companion animals, but no such rules have been issued to date. In the USA, no national regulations on breeding were identified.

### 3.2. Reproductive Limitations (Minimum Age for Mating and Number of Litters)

Age limits and limits on the number of litters (typically per year) may protect the welfare of bitches used for breeding. In England, Italy, the Netherlands, New South Wales, and Sweden, the legislation contains such limits. In New Zealand, limits are recommended in best practice guidance. Austria, Denmark, Germany, and the USA have no legislation on these matters.

Legislation on the minimum age for arranged mating can involve two criteria: a fixed time limit (12 months in England, 18 months in Sweden) and the prohibition of mating during the first oestrous cycle. Both criteria are used in New Zealand’s guidelines. In Sweden, and similarly in New Zealand, it is recommended that a veterinarian should assess whether breeding is advisable when a bitch is older than seven years.

Limits on the frequencies of litters a bitch should have are comparable across the jurisdictions: they are either one per year or, more commonly, two over a period of two years. In New Zealand, the limit on numbers of litters is recommended best practice. The legislation in Sweden and New South Wales allows derogation where this is authorised by a veterinary practitioner. In England, it is prohibited for a bitch to have more than six litters in total over her entire lifespan. Italy has a similar maximum of seven litters in total.

### 3.3. Sales (Point of Sale, Minimum Age Limit for Puppies Being Sold, and Mandatory Care Instructions)

All the European jurisdictions except Germany and Italy have legislation governing (and in some cases prohibiting) the sale of dogs in markets, shops, and the like. No such rules were found in the jurisdictions surveyed outside Europe. In England, dogs may only be sold where they are bred by the breeders, and only when the buyer is present at the seller’s location. Austrian law alone regulates the sale of dogs over the internet, where it requires the seller to have a permit for the commercial sale of dogs.

All jurisdictions have age limits for puppies on sale. In all of them, except the Netherlands and Norway, puppies are required to be eight weeks old before being sold legally (but note that in the USA, this age limit only applied in approximately half of the states). The Netherlands set a minimum age of seven weeks. In Norway, the eight-week age limit serves only as a recommendation. In New Zealand, puppies must be able to feed independently and be in good health when they are made available for sale or rehoming. It is also recommended that puppies should be at least eight weeks of age, have begun socialisation with other dogs and humans, and be completely weaned on to solid food when they are made available for sale or rehoming. In Sweden, derogation from the eight weeks requirement is permissible if authorised by a veterinarian.

Austria, Denmark, England, Italy, the Netherlands, New South Wales, and Norway all require sellers to provide buyers with information on how to take care of the dogs and ensure their welfare. In some jurisdictions (e.g., Denmark), this requirement may be restricted to what are viewed as commercial sales (in the case of Denmark: when the seller uses three or more breeding bitches for producing three or more litters of puppies per calendar year, or in case of resale). In New Zealand, there is no legal requirement to provide the buyer with information. However, the Code of Welfare contains recommendations on the sort of information the seller should supply buyers with.

### 3.4. Surgical Interventions (Neutering, Tail Docking, Ear Cropping, and Debarking)

Surgical interventions such as neutering, tail docking, ear cropping, and the removal of a dog’s vocal cords are regulated across the European jurisdictions. This probably reflect the fact that all of the European countries we examined, except the Netherlands and the UK, have ratified the 1987 Council of Europe Convention for the Protection of Pet Animals, which prohibits surgical interventions intended to alter the appearance of a pet or to have other irreversible purposes. Despite not being party to the Convention, England and the Netherlands also have prohibited such interventions. The legislation applied in New South Wales, New Zealand, and the USA, does not contain a similar general prohibition.

Despite the general prohibition, in the Council of Europe Convention, on surgical interventions without a veterinary or other legitimate purpose, the Convention does allow interventions undertaken to prevent pets from reproducing. With the exception of Norway, all of the selected jurisdictions, including the non-European jurisdictions, allow the neutering (castrating or spaying) of dogs without veterinary justification.

Several European jurisdictions have modified the general prohibition on surgery without a veterinary justification to permit tail docking in some cases. In Denmark, if the puppy is not more than four days old, tail docking is legal for five specific breeds, all customarily used for hunting. In Germany, an exemption from the general ban exists for hunting dogs, and in England (which is not, as part of the UK, a signatory to the Council of Europe Convention), tail docking is allowed on working dogs and on hunt, point, retriever breeds, spaniels, and terriers. In New South Wales, tail docking is only allowed for welfare reasons (typically in response to damage, disease, or other abnormality), and in New Zealand, it is only permitted for the purpose of responding to an existing disease or injury. In the USA, tail docking is not prohibited.

Ear cropping without veterinary justification is prohibited in all the selected European countries, including those that have not ratified the Council of Europe Convention. New South Wales and New Zealand also prohibit ear cropping, but we were not able to identify any such prohibition in the USA.

In all of the European countries we investigated, debarking, or the surgical removal of vocal cords, is prohibited if it has no veterinary justification, but in the USA, it is allowed in several states. New South Wales and New Zealand occupy a middle position. In New South Wales, the operation may only be carried out if an authorised officer has issued an order that requires the dog owner to prevent the dog from engaging in nuisance barking, if the owner has, without success, taken all other reasonable steps such as behavioural training or caging, and if the dog would need to be killed if the vocal cords were not removed. In New Zealand, debarking is permitted only where other suitable means of treating inappropriate barking have been attempted and have failed.

### 3.5. Day-to-Day Handling by Owner (Design of Indoor and Outdoor Environment, Dog Walking, Duration of Time Spent Alone, Use of Collars)

We use the term ‘day-to-day handling’ to refer a sub-field of companion dog welfare covering private practices within the home and when the dog is taken outside, typically, of course, for walks.

The jurisdictions we examined displayed substantial variation in the extent to which this aspect of private dog ownership is regulated.

Austria, Germany, and Sweden have the most extensive legislation relating to the design of the dog’s indoor and outdoor environment. Sweden also has rules concerning the length of time that dogs can be left alone at home as well as minimum requirements for dog walking. The German legislation requires owners to give dogs sufficient exercise in the open air as well as sufficient contact with their caregiver. The nature and extent of exercise and social contact must be adjusted in view of the breed, age, and state of health of the dog. The German Ministry for Food and Agriculture has announced a legislative initiative that will specify the requirement concerning exercise in the open air to at least two daily trips totalling one hour [10].

The legislation operating in Italy, the Netherlands, and New Zealand contains some general requirements laid down in their respective general laws on animal welfare, while the legislation in Denmark, New South Wales, and the USA sets no requirements on how private owners must house their dogs or give them exercise.

With the exception of England, Italy, and the USA, all of the countries we examined have rules limiting prolonged restraint of dogs. In relation to the training of dogs, all of the European countries, as well as New South Wales, already have legislation or are in the process of introducing a ban on the use of electric collars. However, electric collars are not banned in New Zealand and the USA.

### 3.6. Killing (Including Euthanasia)

The killing of unwanted dogs is permitted in all of the countries we studied except Austria, Germany, Italy, and the Netherlands. In the latter countries, it is permitted only where there are justifying reasons such as incurable disease or serious behavioural problems that make the dog dangerous. In the Netherlands, killing due to behavioural problems is allowed only if the problem cannot be solved through behavioural advice for the owner and training. In Denmark, Germany, and most states in the USA, the killing must be carried out by a veterinarian or another person trained for this task (e.g., a person with a hunting license). In Denmark, this requirement does not apply to puppies younger than one week. In Austria, Italy, and the Netherlands, the killing must be carried out by a veterinarian, whereas in England and New South Wales, a similar requirement for a veterinarian or a person authorised by the veterinarian to carry out the killing only applies to dogs kept for breeding or sale.

### 3.7. Overall Comparison

Table 1 presents a comparison of the countries we investigated. The numbers in the table are indicative of our overall assessment of the degree of legislative regulation in the different jurisdictions, as described in Section 2.3.

An example showing how the indicative numbers in a specific welfare category were determined may be helpful at this point. In the case of day-to-day handling, we assigned Germany and Sweden a 6, because they are among the countries with the most extensive legislation regarding the design of the dog’s indoor and outdoor environment, and because both countries have already introduced, or are planning to introduce, minimum requirements for dog walking. Furthermore, both jurisdictions—like most of the other jurisdictions—have rules limiting prolonged restraint and prohibiting the use of painful collars. In Austria and the Netherlands, the regulation of the day-to-day handling is less restrictive. In Austria, this is because there is no minimum requirement on activity for dogs, and in the Netherlands, it is because there are no requirements relating to the physical environment. These two jurisdictions were therefore assigned a 4. The remaining jurisdictions, except the USA, have some rules (in particular, on prolonged restraint and the use of painful collars), but these are quite limited. Despite a certain amount of variation here, we elected to give all of these countries a 2. We gave the USA a 0 to reflect its lack of rules in this welfare category.

In determining the indicative numbers, we took into account any planned initiatives we had identified, whether or not they had been carried out at the time of writing.

Some jurisdictions have developed guidelines, or recommendations, that are not legally binding, such as New Zealand’s recommendation on the age limit for mating and numbers of litters per year, Norway’s guidance on the minimum age of puppies when they are sold, and the rules on dog exercise in several jurisdictions. We reflected this difference between legal and sub-legal rules by dividing the indicative number associated with the latter by two.

In a similar way, the indicative number associated with the regulation of puppy sales in the USA was also divided by two because the age limit governing puppy sales there only applied in approximately half of the country’s states.

Totals of the points, across the various welfare categories, were obtained by simple addition. The different categories were not given differentiated weightings.

## 4. Discussion

As the table above shows, across the eleven Western jurisdictions we examined, there was substantial variation in both the scope and specificity of the regulations applying to companion dogs.

Much of the regulation we identified—e.g., restrictions on breeding and the limits to surgical interventions without veterinary justification—was clearly linked to dog welfare. On the other hand, it is possible to question the link to welfare in other areas, especially with regard to the regulation of killing.

The exactitude, and (as it were) the accuracy, of the ratings in Table 1 can be questioned, but some results and overall tendencies are, we believe, sufficiently clear and well established. First, the USA stands out as having a very low level of regulation generally in this area. The level also appears to be well below average in Denmark and Norway. Conversely, the level of regulation is highest in some of the European countries (Austria, Germany, Italy, Sweden, and the Netherlands) and New South Wales. England and New Zealand are very close to average. We shall now discuss the differences we found in more detail.

### 4.1. Different Approaches to Different Aspects of Dog Welfare

Whether the breeding of dogs with hereditary diseases or other defects should be permitted appears to be a principled and dichotomous issue: either such practices should be allowed or they should not. In reality, however, there might be a significant variation in the way, and the degree to which, the general rules on such breeding are administered. Germany and the Netherlands have commissioned expert opinions which, in various ways, specify criteria and the defects that can be referred to in assessing whether breeding should be allowed. Other jurisdictions rely on general rules which, even when everything else is equal, are more difficult to apply. In Table 1, this is reflected in the 6 assigned to countries that have specified the legal implications of the general rules and the 4 given to jurisdictions that rely solely on a general prohibition (New South Wales was given a 4 because it requires the bitch and male dog to be physically and mentally healthy). Only Denmark and the USA were found to have no regulations on this welfare aspect.

The issue of killing dogs without a justifying reason such as an incurable disease or a serious behavioural problem appears to be even more a question of principle than the issue of breeding on dogs with hereditary diseases or other defects. We reflected this by giving the indicators the values 0 or 6 (since the requirements concerning the execution of the killing are similar across those jurisdictions where killing without a justifying reason is permitted, a further differentiation was not found to be relevant from a comparative perspective). It is interesting, we think, that, among the selected jurisdictions, only Austria, Germany, Italy, and the Netherlands prohibit killing without veterinary reason, whereas the Scandinavian countries and jurisdictions belonging to the Anglosphere have no such prohibition. Beyond the ethical questions relating to the killing of dogs without a justifying reason, the significance of such practices with regard to the general welfare of dogs is, however, not univocal. From a welfare perspective it may be argued, for instance, that dogs left in crowded shelters without any real prospect of ever being welcomed into a new home do not benefit from being left alive. An absence of a ban thus does not necessarily imply that the country in question has not made a deliberate decision on the matter. Rather, the existence of rules on how and by whom obviously reflect that the matter—and the implications for the welfare of dogs—have been the subject of deliberations.

A similar argument may be made for allowing tail docking for some hunting dog breeds, as some studies have found an increased risk of tail damage on these breeds when used for hunting (see ref. [11,12], but for a contrary argument [13]).

Where sales and surgery are concerned, all of the selected jurisdictions apart from the USA have high degrees of regulation, though some European countries have not completely embraced the ban on tail docking laid down in the 1987 Council of Europe Convention for the Protection of Pet Animals. Both sales and surgery necessarily involve at least two parties: a seller and a buyer or an owner and a veterinarian. In both cases, a monetary transaction takes place. Perhaps these aspects serve as a particularly strong incentive for states to intervene. That would explain the widespread regulation (notably, half of the American states also have age limits on the sale of puppies) in these fields.

The last two welfare aspects—i.e., reproductive limitations (age limit for mating and maximum number of litters per year) and the day-to-day handling of dogs—while obviously differing markedly from one another, both also in significant ways differ from the other welfare categories we distinguished. On the one hand, they do not raise questions of principle in the way that the breeding on dogs with hereditary diseases and killing do. On the other, unlike sales and surgery, they will often involve just one party, i.e., the owner (bear in mind that reproduction is a pre-requisite of, but not the same thing as, selling). Thus, perhaps unsurprisingly, these are the aspects of dog welfare regulation where we observed greatest variation. Day-to-day handling by the owner, in particular, was found to be characterised by a high degree of variation (in Section 4.2, we shall discuss this development in more detail.)

At first sight, it appears that a considerable range of welfare-related issues are regulated in the various jurisdictions. It emerges on closer scrutiny, however, that in many of the selected jurisdictions, a number of the issues are regulated either only modestly or not at all. In these cases, the welfare of companion dogs is barely dealt with by the law beyond the protection afforded by general anti-cruelty legislation.

Compared with the specificity of the regulatory frameworks applying to animals kept for other purposes, such as food production and scientific experimentation, the specificity of the regulation applicable to companion dogs appears, in general, to be low. A likely reason for this difference is the following. Laboratory animals, and even more so farm animals, are typically used in a commercial context, or in other contexts where there is an incentive to minimise costs with, potentially, a negative effect on animal welfare. Where companion dogs are concerned, this incentive is less likely to operate (although it could do so, of course, where owners are looking to cut the costs of dog ownership). It may, for instance, be a decisive factor in keeping farm animals in small cages, or restricting their access to outdoor areas, if this appears necessary if the farm is to remain competitive. Commercial considerations of this kind simply do not arise for most private dog owners, although in recognition of the fact that companion dogs are sometimes commercially bred and sold, most jurisdictions have more elaborate regulations on this aspect of canine welfare.

### 4.2. Level of Regulation of Private Practices (Day-to-Day Handling)

Overall, then, there appear to be relatively low levels of regulation protecting the welfare of companion dogs. Various reasons explain the absence of rules in particular welfare areas. With regard to principled questions about, for example, surgical interventions and killing, omission to regulate will most likely be the result of an active decision on whether or not to prohibit a certain practice. Where less-principled questions—e.g., about the day-to-day handling of dogs—are concerned, the absence of regulation can be interpreted differently. Thus, the fact that there are no requirements on, say, design of the indoor and outdoor environment, dog walking, and the duration of time the dog spends alone, does not necessarily imply that the responsible authorities do not care about these issues. Instead of legal requirements, they may find obligatory information to prospective dog owners a more adequate tool.

Beyond this general observation, it is worth noting that there was considerable variation in the levels of regulation (both in terms of scope and specificity) in the countries we examined. The non-European jurisdictions, as well as England (all common law countries), all have low levels of regulation. The picture is more complex in the rest of Europe. Denmark, Italy, and Norway also have a low level of regulation whereas the more central, ‘Germanistic’ European countries (Austria, Germany, the Netherlands) and Sweden have higher levels of regulation, partly reflecting the introduction (or pending introduction) of rules on matters such as time spent outside and physical exercise.

A number of factors may explain these differences. First of all, the introduction of more specific rules applying to what are, in fact, private practices, seems to mirror a trend towards heightened status for companion animals in general and dogs in particular [3]. Variation in the strength of this trend in different countries may account for some differences in the rules. Variation in the rules may also reflect the strength of liberal tradition in the New World countries (the USA, Australia, and, perhaps to a lesser extent, New Zealand), a tradition associated with a particular desire for freedom from the interference of the state.

While these explanations may explain some of the variation, they do not seem to explain why Denmark has a particularly low degree of regulation governing dog ownership in the private sphere relative to its neighbours. In our study, we did not attempt to establish whether this is a peculiarity where companion animals are concerned. For the time being, it thus remains an open question whether, for instance, policy-makers in some countries are of the opinion that the welfare of companion dogs is already sufficient, with no need for further legislative intervention. In this regard, the requirement found in, among other places, Denmark, that the buyer of a dog shall be provided with written information on how to care for it, and ensure its welfare, may be conceived as an alternative and perhaps superior way of ensuring the desired level of welfare for companion dogs than statutory rules, which, indisputably, would be difficult to enforce.

### 4.3. Legal Instruments

Our findings show that three kinds of regulatory technique are being used to regulate dog ownership and encourage suitable behaviour by dog owners: (1) binding legal norms (parliamentary laws and binding administrative regulations such as ministerial orders or binding standards contained in guidelines); (2) non-binding guidelines, codes of conduct, best practice, and the like; (3) non-legal incentives such as instructions that the purchaser of a dog is provided with. There is a clear tendency for the second and third type of behaviour management to be employed more frequently than the first when the issue in question is associated with the private sphere. Thus, in New Zealand, the norms on reproduction (age of bitch and number of litters per year) are construed as non-binding recommendations, and in a majority of the jurisdictions we looked at, dog sellers were required to provide buyers with written instructions holding information on the care and welfare of the dog (in New Zealand, this matter is dealt with in recommendations given in the ‘Code of Welfare’). However, although it is a legal requirement to provide the buyer with instructions, the content of the instructions is not laid down in law. Interestingly, the two countries with the most ambitious, hard-law regulation of the day-to-day handling of dogs, Sweden and Germany, belong to the small minority of jurisdictions having no requirements of written instructions.

The overall picture is thus that issues concerning the handling of dogs in the private sphere tend to be regulated by soft-law, or so-called self-regulation (via the written instructions given to new dog owners). This may well be due to difficulties of enforcement where, e.g., rules on private routines affecting the amount of time that the dog spends alone and the amount of exercise it gets are concerned. Countries that traditionally employ non-binding instruments in situations where enforcement is challenging will presumably be likely to use the same behaviour management tools to manage, for example, the day-to-day handling of companion dogs.

### 4.4. Strengths and Limitations of the Study

This comparative legal examination of the regulation of companion dog welfare in different countries is the first of its kind. We believe it offers a vivid overview.

Our investigation has some limitations. First, by focusing on Western European and other Western jurisdictions, we excluded other parts of the world, including, for example, Eastern European and Asian jurisdictions. The inclusion of these jurisdictions, with their divergent legal traditions and differing cultural and religious attitudes to companion animals, would have permitted a more thorough and complex analysis of the peculiarities of the various legislative domains.

Second, we identified the relevant legislation existing 2020/2021. However, we did not take into account the dates on which the various pieces of legislation in the selected jurisdictions were promulgated. Such information would have not only documented the legal developments within the particular jurisdictions but also allowed us to investigate how the jurisdictions may have inspired or otherwise influenced one another.

It remains possible, of course, that we failed to identify all of the relevant pieces of legislation and guidance in our search for legal texts. It is also possible that the legal documents that were included in the study have subsequently been amended or repealed. We also recognise that, in some cases at least, alternative understandings and interpretations of the legal texts, differing from our own, might reasonably be adopted.

Finally, by only examining the legal norms and not the enforcement of these rules, our study does not give the full picture of the actual effects that the legal requirements will have on the welfare of companion dogs.

## 5. Conclusions

Looking at eleven Western countries, we have demonstrated that, in the developing regulatory frameworks dealing with private dog ownership, significant variation across the jurisdictions exists. This variation concerns both the scope of the regulation and the specificity, or degree of detail, of the regulatory instruments. In addition, the various jurisdictions employ different regulatory tools to achieve the desired outcomes for canine welfare. We suggest there is real potential for mutual sharing and learning in this area.

## Figures and Tables

**Table 1:** Degrees of regulation in a range of welfare categories for companion dogs. Note that the numbers indicating degrees of regulation are not intended to capture the welfare benefit to the dogs.

Country	Breeding (Health of Offspring)	Reproductive Limitations	Sales	Surgical Interventions	Day-to-Day Handling	Killing	Total
Austria	4	0	6	6	4	6	26
Denmark	0	0	6	4	2	0	12
England	4	6	6	4	2	0	22
Germany	6	0	4	4	6	6	26
Italy	4	6	6	6	2	6	30
Netherlands	6	6	6	6	4	6	34
Norway	4	0	3	6	2	0	15
NSW	4	6	6	6	2	0	24
NZ	4	3	6	6	2	0	21
Sweden	4	6	4	6	6	0	26
USA	0	0	2	0	0	0	2
Average	3.6	3.0	5.0	4.9	2.9	2.2	21.6

## Data Availability

Data are available as listed under Supplementary materials (File S1).

## Supplementary Materials

The following materials are available online at https://www.mdpi.com/article/10.3390/ani11061660/s1: File S1: tables listing legislation related to the welfare of companion dogs in 11 countries.
