# Supplementary material for: Regulating Companion Dog Welfare: A Comparative Study of Legal Frameworks in Western Countries
Source: Animals (Basel). 2021 Jun 2;11(6):1660. doi: 10.3390/ani11061660 (PMC8228344; doi:10.3390/ani11061660)
Supplement: Supplementary file 1 [file animals-11-01660-s001.zip › Animals Tables_listing_legislation_related_to_the_welfare_of_companion_dogs_in_11_countries.pdf]

UNIVERSITY OF COPENHAGEN
DEPARTMENT OF FOOD AND RESOURCE ECONOMICS
DEPARTMENT OF VETERINARY AND ANIMAL SCIENCES
CENTRE FOR COMPANION ANIMAL WELFARE

Tables listing legislation related to the welfare of companion dogs in 11 countries

Supplementary material for, Søren Stig Andersen, Iben Meyer, Björn Forkman, Søren Saxmose Nielsen and Peter Sandøe, Regulating companion dog welfare: A comparative study of legal frameworks in Western countries, ms. submitted to *Animals* 30 April 2021.

For comments, please contact Iben Meyer, iben@dyreadfaerdskonsulenten.dk, Søren Stig Andersen, ssa@ifro.ku.dk, or Peter Sandøe, pes@sund.ku.dk.

Frederiksberg, Denmark, April 2021

Content

Tables on legislation related to the welfare of companion dogs in 11 countries 1

Table providing an overview of the legislation in the 11 countries 5

Detailed tables of the legislation in the 11 countries 10

1. Breeding 10
   - 1.1 Table: Breeding 10
   - 1.2 Links: Breeding 12
2. Commercial keeping, breeding and sale 14
   - 2.1 Table: Commercial keeping, breeding and sale 14
   - 2.2 Links: Commercial keeping, breeding and sale 20
3. Dangerous dogs 22
   - 3.1 Table: Dangerous dogs 22
   - 3.2 Links: Dangerous dogs 30
4. ID, registration, insurance and taxes in relation to dog keeping 33
   - 4.1 Table: ID, registration, insurance and taxes in relation to dog keeping 33
   - 4.2 Links: ID, registration, insurance and taxes in relation to dog keeping 37
5. Specific requirements for dog management and physical environment for dog keeping 40
   - 5.1 Table: Specific requirements for dog management and physical environment for dog keeping 40
   - 5.2 Links: Specific requirements for dog management and physical environment for dog keeping 46
6. The dog in the public 48
   - 6.1 Table: The dog in the public 48
   - 6.2 Links: The dog in the public 51
7. Surgery and killing 53

7.1 Table: Surgery and killing 53
7.2 Links: Surgery and killing 57
8. Import 59
8.1. Table: Import 59
8.2. Links: Import 63
9. Stray dogs 65
9.1 Table: Stray dogs 65
9.2 Links: Stray dogs 69

Table providing an overview of the legislation in the 11 countries

Legislation \ Country	Denmark	Sweden	Norway	England[1]	The Netherlands	Germany[2]	Austria[3]	Italy	USA[4]	Australia - New South Wales	New Zealand[5]
**Breeding**											
Bitch minimum age		X		X				X		X	
Limit to number of litters		X*		X	X			X		X	
Breeding restrictions for some breeds due to health issues	(X)	(X)	(X)	(X)	X	X	(X)	(X)		(X)	(X)
**Commercial keeping, breeding and sale**											
Minimum age at sale	X	X*		X	X	X	X	X	X	X	
Sale on physical markets forbidden	X	X	X	X	(X)*		(X)				
Written care instructions required upon sale	X				(X)		X	X		X	
License from authorities required	X	X	(X)	X	(X)	(X)	(X)	(X)			
Requirements for commercial dog keeping	X	(X)	(X)	X	X	X	X	(X)		X	X
Educational requirements for commercial breeders/sellers	X	X	(X)	(X)	(X)	X	(X)	X		X	

Legislation \ Country	Denmark	Sweden	Norway	England[1]	The Netherlands	Germany[2]	Austria[3]	Italy	USA[4]	Australia - New South Wales	New Zealand[5]
**Dangerous dogs**											
Breed ban	X		X	X*		a, b, c	a, b, c		X		
Orders on the use of leash, muzzle, enclosure after dangerous behaviour	X		X	X		a, b, c	a, b, c	X	X	X	X
Killing (euthanasia) orders after dangerous behaviour	X	X	X	X		a, b, c			X	X	X
**ID, registration, insurance and taxes in relation to dog keeping**											
ID required	X	X		X	X	a, b, c	X	X		X	X
National dog register with registration duty	X	X		X	X	b	X	X		X	X
Dog tag/ID required	X			X*	X	a			X	X	X
Compulsory liability insurance	X					b	a, b				
Dog fee/tax					X	a, b, c	a, b, c	X*	X	(X)	X
**Specific requirements for dog management and physical environment for dog keeping**											

Legislation \ Country	Denmark	Sweden	Norway	England[1]	The Netherlands	Germany[2]	Austria[3]	Italy	USA[4]	Australia - New South Wales	New Zealand[5]
Requirements for cage/room size for the dog		X		(X)	X	X	X	(X)			X
Limits for time alone		X									
Walking requirements		X						(X)			X
Ban against tying up dogs for longer periods of time	X	X*			X*	X*	X			(X)	X
Car transport requirements	(X)	X	(X)	X		(X)	(X)	(X)	(X)	(X)	X
Dog doping forbidden			(X)			(X)	(X)	X			
Painful collars forbidden	X	X	X*		(X)	(X)	(X)	(X)		(X)	X*
**The dog in the public**											
Leaving a tied up dog forbidden			X								
Leash requirement in cities	X*				X	a,b,c	a,b,c	X	X	X	X
Muzzle requirement in cities							a,b,c	X			
**Surgery and killing**											
Neutering (without veterinary cause) allowed	X	(X)		X	X	(X)	(X)	X	X	X	X

Country / Legislation	Denmark	Sweden	Norway	England[1]	The Netherlands	Germany[2]	Austria[3]	Italy	USA[4]	Australia - New South Wales	New Zealand[5]
Vaccination requirements											
Tail docking forbidden	X*	(X)	(X)	X*	(X)	(X)*	(X)	X	X	X	X
Ear cropping forbidden	X	(X)	(X)	(X)	(X)	(X)	(X)	X	X	X	X
Removal of vocal cords forbidden	X	(X)	(X)	(X)	(X)	(X)	(X)	X			X*
Ban against killing without proper reason					X	(X)	(X)	X			
Only veterinarians can euthanise dogs					X		(X)	X			
**Import**[¤]											
EU rules for import from third countries as well as trade between EU countries	X	X	(X)	(X)	X	X	X	X			
Puppies can be exempted from the rabies vaccine requirement in connection to trade within the EU	X	X*	X*				X				
Other requirements than the EU rules	X	X	X	X	X		X		X	X	X
Ban against import of certain breeds	X		X	X		X				X	X

Legislation \ Country	Denmark	Sweden	Norway	England[1]	The Netherlands	Germany[2]	Austria[3]	Italy	USA[4]	Australia - New South Wales	New Zealand[5]
**Stray dogs**											
Educational requirements for animal shelter staff	X	X	(X)	(X)	(X)	(X)	X		(X)	(X)	
Requirements for layout of animal shelters	X	X	(X)	(X)	(X)		X	X		(X)	X
Time limit for rehoming/ killing	X	X	X		X		X	X	X	X	X

X Legal texts include information about dogs in relation to the specific subject

(X) Legal texts include information about dogs in relation to a related subject or about animals in general in relation to the specific subject

* There are exemptions from the rule

[1] Referring to the legislation of England. Even though part of the legislation is applied in the UK, in some cases the legislation of Northern Ireland, Scotland and Wales differs from what is presented here.

[2]The Xs refer to the German federal legislation. [a,b,c] indicate whether there is a separate legislation within the area in question in the three selected federal states ([a] Schleswig-Holstein, [b]Hamburg, [c]North Rhine-Westphalia).

[3]The Xs refer to the Austrian federal legislation. [a,b,c] indicate whether there is a separate legislation within the area in question in the three selected federal states ([a] Vienna, [b]Tirol, [c]Vorarlberg).

[4]The Xs indicate that some states or counties have legislation within the area. Detailed differences between the states can be found in the document “Elaboration of dog welfare legislation across countries”.

[5]Parts of the legislation about dogs in New Zealand are provided in the “Code of Welfare: Dogs” where minimum requirements (punishable if not complied with) as well as recommendations for best practice are stated. Only the actual legal rules are included in this table, while the document “Elaboration of dog welfare legislation across countries” includes both minimum requirements as well as best practice recommendations.

[¤] Import covers commercial dog import from third countries (list or non-list countries). See “Detailed tables of the legislation in the 11 countries” for information about the EU rules for import and trade.

Detailed tables of the legislation in the 11 countries

1. Breeding

1.1 Table: Breeding

	Bitch minimum age	Limit to number of litters	Breeding restrictions for some breeds due to health issues
**Denmark**	No legislation identified.	No legislation identified.	The Minister for Food, Agriculture and Fisheries can establish rules for breeding companion and hobby animals, including a ban against such breeding.
**Sweden**	A bitch must be at least 18 months old before the first mating can take place.<br>If a bitch is more than seven years old, a veterinarian must assess whether it is suitable for having puppies.	If a bitch has had two litters within 12 months, she must be given a break of at least 12 months before the next litter. Exceptions can be made if a veterinarian assesses that the welfare of the animal will not be compromised.	Male dog and bitch must be physically and mentally healthy.<br>The breeding of dogs with defects or features which may be inherited and cause distress or unnatural behaviour in the offspring, is forbidden.
**Norway**	No legislation identified.	No legislation identified.	Pertaining to animals in general: The breeding of animals with defects or features which may be inherited and cause distress or unnatural behaviour in the offspring, is forbidden.
**England**	A bitch must be at least 12 months of age before the first mating can take place.	A bitch cannot have more than one litter within 12 months.<br>The bitch cannot have more than six litters in total.<br>A bitch cannot mate again if she has undergone two caesarean sections.	Breeding of dogs, where genotype, phenotype or health status is expected to have a harmful impact on the health or welfare of the dog or the offspring, is forbidden.
**The Netherlands**	No legislation identified.	A bitch cannot have more than one litter within 12 months.	Companion animals cannot be bred in a way that is disadvantageous for the welfare and health of the parent animal or the offspring.<br>Brachycephalic dogs that do not meet certain criteria cannot be bred.
**Germany**	No legislation identified.	No legislation identified.	Pertaining to animals in general: The breeding of individuals with defects or features, which may be

			inherited and cause distress or unnatural behaviour in the offspring, is forbidden.<br>In the interpretation of Article 11b of the animal welfare act, the breeding of individuals of certain breeds is forbidden if they have extreme brachycephaly, “blue dog symdrome”, a short/missing tail, hip joint dysplasia etc.
**Austria**	No legislation identified.	No legislation identified.	Pertaining to animals in general: Breeding, which can be expected to cause pain, distress, harm or fear in the animal or its offspring, is forbidden. Examples mentioned in the legislation are deformities, dyspnoea etc.
**Italy**	Bitches cannot be mated until after their second heat. After seven years, a veterinarian must be consulted prior to breeding.	A bitch should not have more than five litters.	The breeder must strive towards breeding healthy dogs that are free from disease and do not carry any genetic disorders.<br>Dogs which cannot mate naturally should not be inseminated.<br>The breeding of dogs with abnormal behaviour must be avoided.
**The USA**	No legislation identified.	No legislation identified.	No legislation identified.
**Australia**	New South Wales<br>Bitches cannot be mated during their first heat.	New South Wales<br>A bitch cannot have more than two litters over a two year period. Exceptions can be made by means of a written approval from a veterinarian.	New South Wales<br>Dogs must be physically and mentally healthy, well-functioning and free from disease at the time of the mating.
**New Zealand**	No minimum requirements.<br>Best practice recommends that the bitch is not mated until 12 months after its first heat. Furthermore, a veterinarian should be consulted if a bitch is more than seven years old, or if a bitch aged five years or more is mated for the first time.	No minimum requirements.<br>Best practice recommends that bitches are not mated in two successive heats. Only dogs who are in good physical shape should be used for breeding.	Breeders must do their best to ensure that the genetics of the bitch and the male dog do not increase the frequency or degree of a known hereditary condition.

1.2 Links: Breeding

Denmark:

- Lov om dyrevelfærd https://www.retsinformation.dk/eli/lta/2020/133

Sweden:

- Statens jordbruksverks föreskrifter och allmänna råd om hållande av hundar och katter https://www.hsb.se/contentassets/9b52bd940f684adab136bbc99f7b42f2/kalla-foreskrift-om-nya-regler-for-hundar-och-katter-sjvfs2020-8.pdf

Norway:

- Lov om dyrevelferd https://lovdata.no/dokument/NL/lov/2009-06-19-97

England:

- The Animal Welfare (Licensing of Activities Involving Animals) (England) Regulations 2018 https://www.legislation.gov.uk/uksi/2018/486/contents

The Netherlands:

- Besluit houders van dieren https://wetten.overheid.nl/BWBR0035217/2018-07-01
- https://www.rvo.nl/onderwerpen/agrarisch-ondernemen/dieren/dierenwelzijn/welzijnseisen-voor-dieren/regels-voor-fokken
- https://www.fecava.org/wp-content/uploads/2020/06/breeding-short-muzzled-dogs-in-the-netherlands_expertisecentre-genetics-of-companionanimals-2019-translation-from-dutch.pdf
- https://www.fecava.org/wp-content/uploads/2020/06/statement-criteria-short-muzzled-dogs-remain-unchanged-dkc-may-2020.pdf

Germany:

- Tierschutzgesetz http://www.gesetze-im-internet.de/tierschg/BJNR012770972.html
- Gutachten §11b des tierschutzgesetzes https://www.bmel.de/SharedDocs/Downloads/DE/_Tiere/Tierschutz/Gutachten-Leitlinien/Qualzucht.pdf?__blob=publicationFile&v=2

Austria:

- Bundesrecht konsolidiert: Gesamte Rechtsvorschrift für Tierschutzgesetz https://www.ris.bka.gv.at/GeltendeFassung.wxe?Abfrage=Bundesnormen&Gesetzesnummer=20003541

Italy:

- Regolamento Affissi https://www.enci.it/allevatori/regolamento-affissi
- Regolamento Affissi https://www.enci.it/media/7302/regolamentoaffissi.pdf

The USA:

- Personal communication with Andrew Rowan, PhD, and Lisa Moses, VMD, DACVIM
- https://www.animallaw.info/article/detailed-discussion-commercial-breeders-and-puppy-mills-0

Australia:

New South Wales

- NSW Breeding Code https://www.dpi.nsw.gov.au/animals-and-livestock/animal-welfare/general/codes-of-practice/breeding-dogs-and-cats

New Zealand:

- Code of welfare: Dogs https://www.mpi.govt.nz/dmsdocument/1428-Dogs-Animal-Welfare-Codes-of-Welfare

2. Commercial keeping, breeding and sale

2.1 Table: Commercial keeping, breeding and sale

	Minimum age at sale	Sale on physical markets forbidden	Written care instructions required upon sale	License from the authorities required	Requirements for commercial dog keeping	Educational requirements for commercial breeders/sellers
**Denmark**	8 weeks.	It is forbidden to sell dogs at markets.	The legislation states, specifically for the sale of dogs, which subjects these guidelines must cover.	The following is considered commercial and requires a license:<br>● Dog trade (purchasing dogs with the aim of resale)<br>● Breeding (three or more litters per year using three or more breeding bitches)<br>● Operation of dog kennels or shelters with four dogs or more at a time<br><br>The authorities do inspections and publish the results at www.findhundesmiley.dk	Commercial dog keeping is subject to requirements within the following areas:<br>● Prevention of disease<br>● Hygiene<br>● Records<br>● Layout of the premises<br>● Feeding and water<br>● Physical and mental stimulation<br>● Walking<br>● Human contact<br><br>The guidelines for meeting these requirements are very specific.<br><br>Furthermore, there are requirements for the physical surroundings and for the surveillance of watch and guarding dogs.	The person responsible for the business must have completed an education with focus on dog behaviour, care, legislation etc. The act states the subjects to be covered in this education.<br><br>The Danish Veterinary and Food Administration approves the education.<br><br>An education may be replaced by documentation of appropriate experience within the area.

**Sweden**	8 weeks, but exemptions can be made if the puppies are on solid food and their health and welfare (assessed by a veterinarian) are good.	It is forbidden to sell dogs in zoo shops, at markets or the like.	No legislation identified.	The following is considered commercial and requires a license:<br>● Keeping 10 or more dogs over the age of 12 months<br>● Breeding three or more litters per year<br>● Fostering out (e.g. of breeding or working dogs) three or more dogs per year<br>● Sale of dogs from three or more self-bred litters per year<br>● Sale of dogs from three or more litters bred by others per year<br>● Keeping four or more dogs for care or rehoming reasons	No requirements specifically for commercial dog keeping.<br>Requirements for dog keeping in general:<br>● Meeting the dog's physical and mental needs<br>● Cage and room size<br>● Hygiene<br>● Feeding and water<br>● Layout of the premises<br>● Surface, shelter, a place to lie/a kennel for outdoor keeping<br>● Walking the dogs<br>● Human contact<br>The guidelines for meeting these requirements are very specific.	Anyone engaged in commercial dog keeping should have adequate competencies acquired through education or similar experience.<br>The competencies must include the areas of dog behaviour, care, legislation etc.
**Norway**	No legislation identified.<br>Dog guidelines from Mattilsynet (the Norwegian Food Safety Authority) state eight weeks as the minimum age for sale of puppies.	Live animals cannot be displayed in windows. Dogs cannot be sold in/at shops, stalls, markets and the like.	No legislation identified in relation to the sale of dogs.<br>No requirements for written information in relation to the sale of animals.<br>When selling or turning over animals to others, necessary information about matters pertaining to the welfare of the animal must be disclosed.	For animals in general, the following requires a license when it has a commercial aim:<br>● Sale, rent and lending of animals<br>● Kennel<br>● Walking or training the animals of others<br>● Day-care<br>● Companies rehoming animals (including non-profit companies)	The following requirements apply to sale and temporary keeping of animals in general:<br>● Records<br>● Layout of the premises<br>● Surface and shelter for outdoor keeping<br>● Prevention of disease<br>● Feeding and water<br>● Hygiene<br>● Consideration for the animal's behaviour,	When selling and temporarily keeping animals, the person responsible for the company must be able to document the knowledge and skills necessary to manage animal welfare.

					socialisation, health and age<br>● Safety in connection to fire<br><br>Mattilsynet also provides more specific guidelines for dogs.	
**England**	8 weeks	Animals cannot be sold at markets.<br>Dogs can only be sold from the establishment where they were born and bred.<br>It is forbidden to sell puppies who have not been bred by the person holding the license to sell.<br>Dogs can only be sold when the buyer is present at the location of the seller.<br>Puppies can only be presented to potential buyers if their mother is present.	The selling party must make sure that the buyer receives information about feeding, handling, care etc. Appropriate information must be provided to the buyer.<br>The person selling a dog must provide information about the dog's age, sex and veterinary record - no requirement for this information to be in writing.	The following requires a license:<br>● Sale of pets<br>● Caring for the dogs of others with a commercial aim<br>● Dog breeding (3 or more litters within 12 months -unless no dogs are sold - and/or dog breeding and advertising of dog sale)	There are requirements for the following areas in connection to a dog breeding permit:<br>● The license must be visible<br>● Records<br>● Layout of the premises<br>● Feeding and water<br>● Meeting the dog's physical and mental needs<br>● Socialisation<br>● Protection against pain, distress, injury and disease	The person who has obtained the license as well as employees caring for the animals must have the competencies to identify normal behaviour and recognise, prevent and handle signs of pain, distress, injury, disease or unusual behaviour.
**The Netherlands**	7 weeks.	Companion animals cannot be displayed in a shop window.<br>Companion animals can be sold at markets if specific requirements for reporting, veterinary inspection etc. are met.	Pertaining to the sale of animals in general, the seller must provide written information about how to care for the animal.	Pertaining to animals in general, the following is considered commercial and requires registration:<br>● Selling, keeping, breeding with the aim of financial profit<br>● Selling animals<br>● Keeping animals with sales aim<br>● Breeding with sales aim	Commercial keeping, sale and breeding of dogs are subject to requirements within the following areas:<br>● Records<br>● Layout of the premises<br>● Health<br>● Socialisation of young animals	Diploma or certificate documenting an education with focus on the animal you work with. The diploma or certificate must be issued from an accredited institution.

				Selling, keeping or breeding more than 20 dogs within 12 consecutive months.		
**Germany**	A puppy cannot be separated from its mother before it is eight weeks old.	No legislation identified.	No legislation identified.	For animals in general, the following requires a license:<br>● Selling animals<br>● Importing animals with sales aim<br>● Keeping animals in animal shelters<br>● Training of protection dog for a third party<br>● Commercial breeding (three or more bitches or three or more litters per year)<br>● Commercial handling of animals<br>● Commercial transport of animals<br>● Training of dogs for a third party or training of dog and owner	Keeping and breeding dogs are subject to requirements within the following areas:<br>● Exercise<br>● Social human contact<br>● More dogs together<br>● An advisor available for every 1-10 dogs (applies only to breeding)<br>● Kennel and shelter for outdoor keeping<br>● Layout of the premises<br>● Layout of run<br>● Tying up (if applicable)<br>● Feed and water<br>● Inspection<br>● Hygiene	Pertaining to dog breeding, an advisor must be available for every 1-10 dogs with puppies. The advisor must be able to document their knowledge and skills.
**Austria**	A puppy cannot be separated from its mother/siblings before it is eight weeks old.	Animals cannot be sold at public places or by means of "door-to-door sale".<br>Public sales promotion in relation to sale of animals - also on the internet - is only allowed for people with a sales license (breeders, animal shelters, companies).	Written information is not required, but generally, the seller must inform the buyer about appropriate keeping and be able to document that such information has been provided. This also applies to breeders selling puppies.<br>When a dog is sold at an animal shelter or a pet shop, the seller is obliged	For animals in general, a license is required to keep animals with sales or breeding aims.	Requirements for dog keeping in general:<br>● Layout of the premises<br>● Social contact<br>● Outdoor keeping<br>● Layout of run<br>● Feed and water<br>● More dogs together<br>The guidelines for meeting these	A sufficient number of people with knowledge about animal welfare must regularly and continuously be active at establishments where animals are kept with sales or breeding aims.

			to provide written information to the buyer.		requirements are very specific.	
**Italy**	Minimum 60 days.	No legislation identified	Breeders must inform the buyer about the breed and about dog owner responsibilities, preferably in writing.	The regions issue licences for commercial keeping, breeding and training of dogs.	The local veterinarian must monitor and inspect premises and equipment used in commercial trade, breeding and training of animals.	Dog breeders must be educated within the breed they are breeding.<br><br>No legislation identified about educational requirements for commercial animal keeping.
**The USA**	Around 28 states have a minimum age for selling puppies/separating puppies from their mother; most commonly eight weeks.<br><br>In 15 of these states, the minimum age applies to all who sell puppies. In the remaining states, it only applies to commercial sale.<br><br>Several states do not have a minimum age.	No legislation identified.	In around 20 states, there are requirements for which information the seller must provide to the buyer of the dog.	Generally speaking, selling animals requires a license. Pet shops and breeders with four or fewer breeding bitches are, amongst others, exempted from this.<br><br>Around 29 states have defined commercial keeping, breeding and/or sale of dogs.<br><br>The number of dogs and/or litters defining whether it is commercial dog keeping/breeding differs greatly from state to state.	The requirements for and number of guidelines pertaining to the layout of the premises of commercial dog keeping/breeding differs greatly from state to state.	No legislation identified.
**Australia**	New South Wales<br>8 weeks.	New South Wales<br>No bans.<br><br>Sales commercials/notices (at markets, in pet shops and via the internet) must include ID number from the NSW Pet Registry, microchip ID, breeder ID or the ID of the rehoming organisation.	New South Wales<br>The buyer must be offered written information about the care of the purchased animal.	New South Wales<br>License not required, but the breeder/seller must meet the requirements of the "Breeding Code" and/or the "Pet Shop Code".	New South Wales<br>Requirements for breeding and selling animals:<br>● Records<br>● Shelter and protection from heat and cold<br>● Places to sleep<br>● Surface<br>● Cage size<br>● Indoor climate	New South Wales<br>With regards to breeding, a person with the ability to ensure that the requirements for care and welfare are met must be present on a daily basis.<br><br>With regards to sale, it is required that the staff is educated within and has

					● Hygiene<br>● Disease prevention (including veterinary inspection)<br>● Killing<br>● Physical and mental stimulation<br>● Feed and water<br>● Transportation<br><br>The guidelines for meeting these requirements are very specific.	experience with animal welfare and care.<br><br>With regards to “boarding establishments” (commercial housing of animals, including veterinary hospitals), it is required that the staff is experienced in handling the animals, and formal training/education is encouraged.
**New Zealand**	No minimum age, but puppies must be able to eat by themselves and be healthy before they can be sold.<br><br>Best practice recommends eight weeks.	No legislation identified.	No legal requirements.<br><br>Best practice recommends that the buyer must be informed about care, training and handling of the puppy as well as about hereditary conditions to which the puppy has a predisposition. No requirements about written form.	No legislation identified.	Requirements for dog keeping in general, including commercial dog keeping:<br><br>● Feed and water<br>● Tying up (if applicable)<br>● A place to lie down<br>● Shelter and protection from heat and cold<br>● Hygiene<br><br>In addition to the compulsory minimum requirements, best practice offers very specific guidelines.	No legislation identified.

2.2 Links: Commercial keeping, breeding and sale

Denmark:

- Bekendtgørelse om dyrevelfærdsmæssige mindstekrav til hold af hunde https://www.retsinformation.dk/eli/lta/2020/1749

Sweden:

- Statens jordbruksverks föreskrifter och allmänna råd om hållande av hundar och katter https://www.hsb.se/contentassets/9b52bd940f684adab136bbc99f7b42f2/kalla-foreskrift-om-nya-regler-for-hundar-och-katter-sjvfs2020-8.pdf
- Föreskrifter om tillståndsplikt för viss djurhållning av sällskapsdjur, häst och pälsdjur http://djur.jordbruksverket.se/download/18.7c1e1fce169bee5214fb19d3/1554448722900/2019-027.pdf

Norway:

- Lov om dyrevelferd https://lovdata.no/dokument/NL/lov/2009-06-19-97
- Lov om hundehold https://lovdata.no/dokument/NL/lov/2003-07-04-74?q=lov%20om%20hund
- Forskrift om omsetning og midlertidig hold av dyr https://lovdata.no/dokument/SF/forskrift/2015-08-11-958
- Tilsynsveileder hund https://www.mattilsynet.no/om_mattilsynet/gjeldende_regelverk/veiledere/tilsynsveileder__hund.16904/binary/Tilsynsveileder%20-%20hund

England:

- The Animal Welfare (Licensing of Activities Involving Animals) (England) Regulations 2018 The Animal Welfare (Licensing of Activities Involving Animals) (England) Regulations 2018 (legislation.gov.uk)
- https://www.gov.uk/guidance/selling-animals-as-pets-licence-england

The Netherlands:

- Besluit houders van dieren https://wetten.overheid.nl/BWBR0035217/2018-07-01
- Wet dieren https://wetten.overheid.nl/BWBR0030250/2020-01-01/#Hoofdstuk2_Paragraaf1_Artikel2.2
- https://www.rvo.nl/onderwerpen/agrarisch-ondernemen/dieren-houden/huisdieren-houden-en-fokken/bedrijfsmatig-huisdieren-houden

Germany:

- Tierschutz-Hundeverordnung http://www.gesetze-im-internet.de/tierschhuv/index.html#BJNR083800001BJNE000100305
- Tierschutzgesetz http://www.gesetze-im-internet.de/tierschg/BJNR012770972.html
- Allgemeine Verwaltungsvorschrift zur Durchführung des Tierschutzgesetzes http://www.verwaltungsvorschriften-im-internet.de/bsvwvbund_09022000_32135220006.htm

Austria:

- Bundesrecht konsolidiert: Gesamte Rechtsvorschrift für Tierschutzgesetz https://www.ris.bka.gv.at/GeltendeFassung.wxe?Abfrage=Bundesnormen&Gesetzesnummer=20003541
- Bundesrecht konsolidiert: Gesamte Rechtsvorschrift für 2. Tierhaltungsverordnung https://www.ris.bka.gv.at/GeltendeFassung.wxe?Abfrage=Bundesnormen&Gesetzesnummer=20003860
- Bundesrecht konsolidiert: Gesamte Rechtsvorschrift für Tierschutz-Sonderhaltungsverordnung https://www.ris.bka.gv.at/GeltendeFassung.wxe?Abfrage=Bundesnormen&Gesetzesnummer=20010231

Italy:

- http://www.salute.gov.it/imgs/C_17_opuscoliPoster_41_allegato.pdf
- Regolamento Affissi https://www.enci.it/allevatori/regolamento-affissi

The USA:

- Personal communication with Andrew Rowan, PhD, and Lisa Moses, VMD, DACVIM
- https://www.aphis.usda.gov/animal_welfare/downloads/AC_BlueBook_AWA_508_comp_version.pdf
- https://www.aphis.usda.gov/aphis/ourfocus/animalwelfare/SA_Regulated_Businesses
- https://www.animallaw.info/topic/table-state-laws-concerning-minimum-age-sale-puppies
- https://www.animallaw.info/article/detailed-discussion-commercial-breeders-and-puppy-mills-0
- https://www.animallaw.info/intro/pet-purchaser-protectionpuppy-lemon-laws
- https://www.animallaw.info/topic/table-state-commercial-pet-breeders-laws

Australia:

New South Wales

- NSW Pet Shop Code - https://www.dpi.nsw.gov.au/animals-and-livestock/animal-welfare/general/codes-of-practice/pet-shops
- NSW Breeding Code - https://www.dpi.nsw.gov.au/animals-and-livestock/animal-welfare/general/codes-of-practice/breeding-dogs-and-cats
- https://www.dpi.nsw.gov.au/animals-and-livestock/animal-welfare/general/welfare-of-dogs/aw-code-5

New Zealand:

- Code of welfare – dogs https://www.mpi.govt.nz/dmsdocument/1428-Dogs-Animal-Welfare-Codes-of-Welfare
- Animal Welfare Act 1999 https://www.legislation.govt.nz/act/public/1999/0142/latest/DLM49664.html?search=ts_act%40bill%40regulation%40deemedreg_animal+welfare+act_resel_25_a&p=1
- Animal Welfare Act 2020 https://www.legislation.govt.nz/regulation/public/2020/0172/latest/LMS329846.html

3. Dangerous dogs

3.1 Table: Dangerous dogs

	Breed ban	Orders on the use of leash, muzzle, enclosure after dangerous behaviour	Killing (euthanasia) orders after dangerous behaviour
**Denmark**	Possession and breeding of the following breeds are forbidden:<br>● Pitbull terrier<br>● Tosa inu<br>● American staffordshire terrier<br>● Fila brasileiro<br>● Dogo argentino<br>● American bulldog<br>● Boerboel<br>● Kangal<br>● Central Asian shepherd dog<br>● Caucasian shepherd dog<br>● South Russian sheepdog<br>● Tornjak<br>● Sarplaninac	If a dog has harmed a human being or caused other significant injuries, or if the behaviour of the dog or the owner instils fear, or if it is reasonable to assume that the dog is dangerous to its surroundings, the police may order the following:<br>● The dog must be kept in an enclosure<br>● The dog must only be walked by its owner or other named persons over the age of 18<br>● When the dog is outside the property, it must be kept on a leash of no more than two metres, wear a muzzle or both	If a dog - in the case of an attack or another unacceptable and dangerous behaviour – has savaged a person or another dog, the dog must be euthanised.<br>The police may also issue a killing (euthanasia) order, if a dog has harmed a human being, or caused other significant injuries, or if the behaviour of the dog, or the owner causes fear in the surroundings, or if it is reasonable to assume that the dog is dangerous to its surroundings.<br>Before the destruction order is carried out, the police or the owner can demand for a dog expert assessment to be conducted. The dog experts are veterinarians with special knowledge about dog behaviour.
**Sweden**	No specific breed bans.<br>It is forbidden to keep or sell cross-breeds between dog and wolf.<br>It is forbidden to keep or breed dogs who:<br>● are excessively aggressive<br>● easily get scared and bite<br>● are difficult to stop in the case of an attack/a fight<br>● attack people and other dogs	No legislation identified.<br>To prevent a dog from causing harm or significant inconvenience, Länsstyrelsen (the County Administrative Board) can decide to remove the dog from its owner. After assessing the dog, the board decides whether the dog can return to its owner or whether it must be rehomed or euthanised.	Dogs who have been removed from their owner must undergo behavioural assessment. If the dog is deemed dangerous to people or animals, it must be euthanised.<br>If a dog is loose in an area with domestic animals and cannot be caught, the owner/carer of the domestic animals is allowed to kill the dog in order to prevent harm to the domestic animals.
**Norway**	It is forbidden to keep, breed or import dogs of the following breeds or cross-breeds including these breeds:<br>● Pitbull terrier<br>● American staffordshire terrier<br>● Fila brasileiro<br>● Tosa inu	If the dog is dangerous or instils fear, the police may issue the following orders:<br>● The dog must be kept on a leash<br>● Temporary use of muzzle<br>● Fence around the property<br>● Reduce the number of dogs<br>● Ban against entering certain areas	Normally, a dog must be euthanised if it:<br>● Significantly harms a child<br>● Attacks or significantly harms production animals or reindeer<br>● Attacks or significantly harms other domestic animals (dogs who are not considered to pose a

	● Dogo argentino<br>● Cross-breeds between dog and wolf<br><br>Furthermore, it is forbidden to keep or import dogs who are trained in attacking people or other dogs or who prove to be particularly aggressive.		larger risk in the future compared to other dogs can be exempted)
**England**	It is forbidden to own, sell, pass on or breed dogs of the following breeds/types:<br>● Pitbull terrier<br>● Japanese tosa<br>● Dogo argentino<br>● Fila brasileiro<br><br>If the police deems the dog not dangerous to the public, it can be included in the "index of exempted dogs" which requires that the dog is:<br>● Neutered<br>● Microchipped<br>● Kept on a leash and wearing a muzzle when in public<br>● Kept in an enclosure<br>● The owner must take out a liability insurance<br>● The owner must carry with them a certificate and show it upon request	If a dog harms people or other people's animals or if it makes others afraid that it might harm them, it is considered "dangerously out of control".<br>For the purpose of securing control over the dog, the owner may be ordered to use a muzzle or a leash, be banned from certain areas or be ordered to neuter a male dog.	A dog of a banned breed (except for individuals who are deemed not dangerous to the public) or a dog who has been "dangerously out of control" in a severe way must be euthanised.<br>A farmer has the right to kill a dog if it frightens their animals.
**The Netherlands**	No breed ban (revoked in 2008).	No legislation identified.	No legislation identified.<br>Animals in general can be euthanised if they pose a danger to people or other animals.
**Germany**	<u>Federal level</u><br>It is forbidden to import (but not to keep) dogs of the following breeds or cross-breeds including these breeds:<br>● Pitbull terrier<br>● American staffordshire terrier<br>● Staffordshire<br>● Bull terrier<br><br><u>Schleswig-Holstein</u>	<u>Schleswig-Holstein</u><br>A dog can be declared "dangerous" based on behaviour (biting a person, repeated incidents with aggressive behaviour in public, biting another animal without being attacked, hunting/killing animals).<br>A permit is required in order to own a "dangerous" dog, and the following requirements apply:<br>● The owner must be over 18 years old<br>● The owner must be deemed reliable and suitable<br>● Owner and dog must pass a 'Sachkundeprüfung'	<u>Schleswig-Holstein</u><br>The authorities may order a "dangerous" dog euthanised if it still poses a danger.<br><u>Hamburg</u><br>The authorities may order a dangerous dog euthanised if it still poses a danger and it is deemed impossible to keep the animal in reasonable conditions with regards to animal welfare.<br><u>North Rhine-Westphalia</u>

	No breed bans.<br><br><u>Hamburg</u><br>It is forbidden to own a “dangerous dog”, but it is possible to apply for a permit.<br><br>A dog can be declared dangerous based on its behaviour.<br><br>The following breeds or cross-breeds including these breeds are always considered dangerous:<br><br>● American pitbull terrier<br>● American staffordshire terrier<br>● Staffordshire bull terrier<br>● Bull terrier<br><br>The following breeds or cross-breeds including these breeds are considered possibly dangerous:<br><br>● Bullmastiff<br>● Dogo argentino<br>● Dogue de Bordeaux<br>● Fila brasileiro<br>● Kangal<br>● Caucasian shepherd dog<br>● Mastiff<br>● Spanish mastiff<br>● Neapolitan mastiff<br>● Rottweiler<br>● Tosa inu<br><br>Legal requirement for annual statistics about biting incidents with information about the dog breeds, district, private/public place, reason for and consequence of the biting incident.<br><br><u>North Rhine-Westphalia</u><br>A permit is required in order to keep a “dangerous dog”. The following breeds or cross-breeds including these breeds are considered dangerous:<br><br>● Pitbull terrier<br>● American staffordshire terrier<br>● Staffordshire bull terrier	● The dog must have an ID<br>● Liability insurance<br>● Securing the property to keep the dog from escaping<br>● The dog can only be walked by its owner in public<br>● Leash order, maximum length of two metres<br>● Muzzle order (for dogs over the age of six months)<br>● The owner must carry with them the permit<br><br>Upon request, the dog may be exempted from the “dangerous” mark after a “Wesenstest” conducted by a person who is approved by the veterinary association of Schleswig-Holstein as well as a subsequent veterinary assessment.<br><br><u>Hamburg</u><br>Owning a “dangerous” dog requires a permit (some breeds as well as dogs who have bitten people or animals, attacked people or hunt and bite deer or other animals).<br><br>Furthermore, the following requirements apply:<br><br>● The owner must have a proper reason to own the dog<br>● The dog is surgically castrated<br>● Liability insurance<br>● The dog must have an ID<br>● The owner and the dog have attended dog training aimed at dangerous dogs<br>● The dog is kept on a leash with a maximum length of two metres<br>● The dog (over the age of nine months) wears a muzzle<br>● Securing the property to keep the dog from escaping<br><br>For owners of “always dangerous” dogs (certain breeds), the following requirements apply:<br><br>● The owner must prove that they are physically and mentally suitable and reliable	The police may order dangerous dogs euthanised if they are deemed posing a danger.

	Dogs of the following breeds (or cross-breeds including these breeds) or size also require a permit:<br><br>● Alano<br>● American bulldog<br>● Bullmastiff<br>● Mastiff<br>● Spanish mastiff<br>● Neapolitan mastiff<br>● Fila brasileiro<br>● Dogo argentino<br>● Rottweiler<br>● Tosa inu<br><br>● Dogs with an adult height exceeding 40 cm<br>● Dogs with a weight of more than 20 kg	● The dog must only be walked by an approved person<br>● There must be at least one other approved person<br>● Documentation of regular training<br>● The dog cannot come from illegal breeding or import<br><br>Dogs who have shown dangerous behaviour or due to their breed are deemed “possibly dangerous” (but not “always dangerous” dogs of certain breeds) can be exempted from the “dangerous” mark if they pass the “Wesenstest”.<br><br>For dogs under the age of 15 months, the exemption is temporary, and the test must be repeated. The test is conducted by a person who specialises in ethology.<br><br>North Rhine-Westphalia<br>A permit is required to own dangerous dogs (certain breeds and dogs who have bitten people, bitten other dogs without being attacked, dogs who have been bred or trained with the aim of aggressiveness, trained for protection or who hunt/harm deer or other animals), dogs of certain breeds as well as large dogs. Conditions for obtaining a permit:<br><br>● The owner must be over 18 years old<br>● The owner must be deemed reliable<br>● Certificate documenting knowledge and skills (Sachkunde) issued by veterinarians and other professions<br>● Liability insurance<br>● Microchipped<br>● Reason for keeping the dog<br>● Securing the property to keep the dog from escaping<br>● The dog must be kept on a leash in public<br>● The dog (over the age of six months) wears a muzzle	
**Austria**	Vienna	Vienna<br>Requirements for dogs over the age of six months for which a license is required:	No legislation identified.

	The following breeds or cross-breeds including these breeds require that the owner completes and obtains a dog certificate/dog license:<br>● Bull terrier<br>● Staffordshire bull terrier<br>● American staffordshire terrier<br>● Neapolitan mastiff<br>● Spanish mastiff<br>● Fila brasileiro<br>● Mastiff<br>● Bullmastiff<br>● Tosa inu<br>● Pitbull terrier<br>● Rottweiler<br>● Dogo argentino<br><br><u>Tirol</u><br>No breed list.<br><br><u>Vorarlberg</u><br>The following breeds or cross-breeds including these breeds require a permit:<br>● Bull terrier<br>● Staffordshire bull terrier<br>● American staffordshire terrier<br>● Neapolitan mastiff<br>● Argentinian dogo<br>● Mastiff<br>● Bullmastiff<br>● Tosa inu<br>● Dogue de Bordeaux<br>● Dogo argentino<br>● Ridgeback<br>● Bandog<br>● Pitbull terrier	● Documentation of dog tax and liability insurance and no criminal record<br>● Passed dog handler course (theory and practice) or documentation for 10 hours with a qualified trainer<br>● The owner must carry with them the dog license when in public<br>● The dog must be kept on a leash in public<br>● The dog must wear a muzzle in public<br>● The owner cannot be under the influence of alcohol (0.5 permille) or drugs when walking the dog<br><br>All dog owners must obtain a dog competence certificate (Sachkundenachweis).<br><br><u>Tirol</u><br>Requirements for dogs who are declared “suspicious” by the official veterinarian:<br>● The dog must be kept on a leash in public<br>● The dog must wear a muzzle in public<br>● The owner may be required to complete training with the dog or have it examined by a veterinarian<br><br>“Suspicious dogs” can only be owned by people who are deemed reliable (i.e. no alcohol addiction, no sentences).<br><br>All dog owners must pass a theoretical course (Sachkundenachweis) the first time they register a dog.<br><br><u>Vorarlberg</u><br>Keeping a listed dog requires the following:<br>● Permit from the dog is 12 weeks old<br>● The dog must be kept on a leash in public<br>● The dog must wear a muzzle in public<br><br>The individual municipalities may have additional rules.<br><br>Participation in a voluntary dog owner course is offered to all dog owners taught by veterinarians who specialise in behaviour.	

**Italy**	No breed list (revoked in 2009).	Dogs requiring behavioural assessment are locally identified and registered. Requirements for these dogs:<br>● The owner must complete a compulsory course developed by veterinarians specialised in behaviour<br>● Liability insurance<br>● Leash and muzzle in urban environments	It is forbidden to euthanise dogs without a legally acceptable reason - e.g. if the dog is incurably ill or a danger to its surroundings.
**The USA**	A downright breed ban is not found in any states.<br>Some counties and cities have breed bans.<br>Some counties have special rules/requirements for dogs of certain breeds.	42 states have legislation about dogs which have shown dangerous behaviour (dangerous dog laws).<br>Some states classify dogs, besides “dangerous”, as “vicious” (more serious) or “nuisance”/“menacing” (less serious)<br>There are differences between states and counties regarding the definition of a dangerous dog. Typically, a dangerous dog is defined by one or more of the following:<br>● Used or trained for dog fighting<br>● Bites or attacks people in public or private places<br>● Bites and harms a person without provocation<br>● Kills a person<br>● Seriously harms or kills other animals<br>● Attacks people in public spaces<br>There are differences between states and counties regarding the orders and restrictions imposed on a “dangerous” dog and its owner. Typically, they include one or more of the following:<br>● The dog is registered as dangerous<br>● Warning sign at the property<br>● Secure enclosure<br>● Leash and/or muzzle when outside of the enclosure<br>● Neutering<br>● Liability insurance<br>● Higher insurance premium of the house - in some cases, it is difficult/impossible to insure the house<br>● ID with tattoo, microchip or special collar<br>● Passing on orders to a new owner (if applicable)	Some states have a legal requirement for or possibility to euthanise dangerous dogs, although this is rarely carried out in practice - instead, the dog is “deported” and can move to another state.<br>There are differences between the states regarding the circumstances leading to killing.

**Australia**	New South Wales<br>It is forbidden to import dogs of the following breeds:<br>● American pitbull terrier or pitbull terrier<br>● Japanese tosa<br>● Dogo argentino<br>● Fila brasileiro<br>● Presa canario<br><br>Dogs of these breeds can be kept in Australia, but certain requirements apply, and the dog is classified as "restricted".	New South Wales<br>A dog can be a "restricted breed" or be declared "dangerous" on the basis of its behaviour (it has attacked or killed a person or another animal without provocation, it is kept for certain types of hunting, it is declared "dangerous" in another area).<br><br>Requirements for keeping a "restricted" or "dangerous" dog:<br>● Annual permit and tax<br>● Microchip and registration<br>● Neutering of the dog<br>● Secure enclosure for the dog on the property as well as warning signs around the property<br>● The dog must be kept on a leash and wear a muzzle in public<br>● The dog must wear a specific collar<br>● It must be reported to the local authorities if the dog attacks or harms people or animals<br><br>Dogs who are cross-breeds of "restricted" breeds can be exempted from the requirements if they pass a behavioural test.<br><br>The owner of a dog who is declared "dangerous" can appeal and must then let the dog undergo behavioural assessment carried out by a professional. The authorities do not provide assessment professionals nor do they provide advice in this regard.<br><br>A dog is classified as "menacing" if it has been excessively aggressive towards a person or another animal or if it without provocation has attacked a person or another animal without harming them.<br><br>Requirements for keeping a "menacing" dog:<br>● The same as for "restricted" and "dangerous" dogs, except regarding the enclosure requirement, since a "menacing" dog must only be in an enclosure if it is not under control by a person.	New South Wales<br>Killing is required if control measures or permanent removal of the dog from the owner do not ensure that the dog does not pose a danger to others. Both dog and owner are assessed in this regard.

**New Zealand**	It is forbidden to import dogs of the following breeds/types or cross-breeds including these breeds:<br>● Fila brasileiro<br>● Dogo argentino<br>● Japanese tosa/tosa inu<br>● Presa canario<br>● American pitbull terrier<br><br>Dogs of these types living in the country are classified as “menacing”.	Dogs can be classified as “dangerous” or “menacing” on the basis of their behaviour.<br>Requirements for keeping “dangerous dogs”:<br>● The dog must be neutered<br>● Secure enclosure of the property<br>● The dog must be kept on a leash and wear a muzzle in public<br>● Higher registration fee<br>● Authority approval is necessary if the dog changes owner<br><br>Requirements for keeping “menacing dogs”:<br>● The authorities may order the dog neutered<br>● The dog must wear a muzzle in public<br><br>In the case of certain violations of the law, the owner may be classified as “probationary owner” which can bring more responsibilities or take away the owner’s right to keep a dog for up to five years.<br>“Probationary owners” can be required to attend a dog training course (the course must be approved by the local authorities).	If a dog causes serious harm to people or kills protected wildlife, the dog must be euthanised.

3.2 Links: Dangerous dogs

Denmark:

- Bekendtgørelse af lov om hunde https://www.retsinformation.dk/eli/lta/2021/329
- Vejledning om hundelovens forbudsordning, regler om skambid mv. https://www.retsinformation.dk/eli/retsinfo/2016/9956
- Bekendtgørelse om dyrevelfærdsmæssige mindstekrav til hold af hunde https://www.retsinformation.dk/eli/lta/2020/1749

Sweden:

- Lov om tilsyn af hund og kat https://lagen.nu/2007:1150#P11S1
- Djurskyddsförordning https://lagen.nu/2019:66

Norway:

- Forskrift om hunder https://lovdata.no/dokument/SF/forskrift/2004-08-20-1204?q=forskrift%20om%20hund
- Lov om hundehold https://lovdata.no/dokument/NL/lov/2003-07-04-74?q=lov%20om%20hunder

England:

- https://www.gov.uk/control-dog-public
- Dangerous Dogs Act 1991 https://www.legislation.gov.uk/ukpga/1991/65/introduction#commentary-c932828
- Dangerous Dogs (Amendment) Act 1997 https://www.legislation.gov.uk/ukpga/1997/53/contents
- Guard Dogs Act 1975 https://www.legislation.gov.uk/ukpga/1975/50/contents

The Netherlands:

- https://business.gov.nl/regulation/licence-private-security-organisation/
- Besluit houders van dieren https://wetten.overheid.nl/BWBR0035217/2018-07-01

Germany:

Federal level

- Tierschutzgesetz http://www.gesetze-im-internet.de/tierschg/BJNR012770972.html
- Gewebeordning §34 Bewachungsgewerbe http://www.gesetze-im-internet.de/gewo/__34a.html

Schleswig-Holstein

- Gesetz über das Halten von Hunden http://www.gesetze-rechtsprechung.sh.juris.de/jportal/?quelle=jlink&query=HuG+SH&psml=bsshoprod.psml&max=true&aiz=true
- schleswig-holstein.de - Inhalte - Private Hundehaltung (schleswig-holstein.de)

Hamburg

- https://www.hamburg.de/hundegesetz
- Hamburgisches Gesetz über das Halten und Führen von Hunden http://www.landesrecht-hamburg.de/jportal/portal/page/bshaprod.psml?showdoccase=1&st=lr&doc.id=jlr-HuGHArahmen&doc.part=X&doc.origin=bs
- Verordnung zur Durchführung des Hundegesetzes http://www.landesrecht-hamburg.de/jportal/portal/page/bshaprod.psml?showdoccase=1&st=null&doc.id=jlr-HuGDVHArahmen&doc.part=X&doc.origin=bs

North Rhine-Westphalia

- Hundegesetz für das Land Nordrhein-Westfalen https://recht.nrw.de/lmi/owa/br_bes_text?anw_nr=2&bes_id=5116&gld_nr=2&ugl_nr=2060&menu=1&sg=0&aufgehoben=N&keyword=hund#det

Austria:

Vienna

- Landesrecht konsolidiert Wien: Gesamte Rechtsvorschrift für Festlegung von hundeführscheinpflichtigen Hunden https://www.ris.bka.gv.at/GeltendeFassung.wxe?Abfrage=LrW&Gesetzesnummer=20000217
- Landesrecht konsolidiert Wien: Gesamte Rechtsvorschrift für Wiener Hundeführscheinverordnung https://www.ris.bka.gv.at/GeltendeFassung.wxe?Abfrage=LrW&Gesetzesnummer=20000209
- Landesrecht konsolidiert Wien: Gesamte Rechtsvorschrift für Wiener Tierhaltegesetz https://www.ris.bka.gv.at/GeltendeFassung.wxe?Abfrage=LrW&Gesetzesnummer=20000404
- Wiener Hunde-sachkundenachweis-Verordnung https://www.ris.bka.gv.at/Dokumente/Gemeinderecht/GEMRE_WI_90101_L200_010_2019/GEMRE_WI_90101_L200_010_2019.pdf
- https://www.oesterreich.gv.at/themen/freizeit_und_strassenverkehr/haustiere/1/2/Seite.742280.html

Tirol

- Landesrecht konsolidiert Tirol: Gesamte Rechtsvorschrift für Landes-Polizeigesetz https://www.ris.bka.gv.at/GeltendeFassung.wxe?Abfrage=LrT&Gesetzesnummer=20000176
- https://www.oesterreich.gv.at/themen/freizeit_und_strassenverkehr/haustiere/1/2/Seite.742260.html

Vorarlberg

- Landesrecht konsolidiert Vorarlberg: Gesamte Rechtsvorschrift für Verordnung der Landesregierung über das Halten von Kampfhunden https://www.ris.bka.gv.at/GeltendeFassung.wxe?Abfrage=LrVbg&Gesetzesnummer=20000252
- https://vorarlberg.at/web/land-vorarlberg/contentdetailseite/-/asset_publisher/qA6AJ38txu0k/content/sachkundenachweis-fuer-hundehalter?article_id=235398

Italy:

- Decreto 26 novembre 2009 https://www.trovanorme.salute.gov.it/norme/dettaglioAtto?id=32050
- Legge 189/2004 https://www.lav.it/cpanelav/js/ckeditor/kcfinder/upload/files/files/Legge%20189-2004.pdf
- http://www.salute.gov.it/portale/temi/p2_6.jsp?lingua=italiano&id=1533&area=cani&menu=tutela
- https://sivemp.it/wp/wp-content/uploads/2019/03/33_46_52_rossi.pdf

The USA:

- Personal communication with Andrew Rowan, PhD, and Lisa Moses, VMD, DACVIM
- https://www.animallaw.info/topic/state-dangerous-dog-laws
- https://www.animallaw.info/article/brief-overview-dangerous-dog-laws

Australia:

New South Wales

- https://www.olg.nsw.gov.au/public/dogs-cats/responsible-pet-ownership/restricted-dogs/
- https://www.olg.nsw.gov.au/public/dogs-cats/responsible-pet-ownership/declared-dangerous-and-menacing-dogs/
- http://www5.austlii.edu.au/au/legis/nsw/consol_act/caa1998174/s48.html

New Zealand:

- Dog control act https://legislation.govt.nz/act/public/1996/0013/latest/whole.html?search=ts_act%40bill%40regulation%40deemedreg_dog_resel_25_a&p=1#DLM374489
- https://www.dia.govt.nz/diawebsite.nsf/wpg_URL/Resource-material-Dog-Control-Dog-Control-Amendment-Act-2003
- https://www.dia.govt.nz/pubforms.nsf/URL/Dog_Enforcement_Guidelines.pdf/$file/Dog_Enforcement_Guidelines.pdf

4. ID, registration, insurance and taxes in relation to dog keeping

4.1 Table: ID, registration, insurance and taxes in relation to dog keeping

	**ID required**	**National dog register with registration duty**	**Dog tag/ID required**	**Compulsory liability insurance**	**Dog fee/tax**
**Denmark**	The dog owner must make sure that the dog carries its ID by means of either microchip or ear or groin tattoo and that it is registered from eight weeks of age at the latest.	The dog owner must make sure that the dog has an ID and is registered from eight weeks of age at the latest.<br>When importing a dog, it must be registered no later than four weeks after arrival.	The owner must make sure that the dog wears a collar with a tag stating the name and address of the owner from the age of four months	Yes.	No.
**Sweden**	The dog owner must make sure that the dog carries its ID via a microchip or an ear tattoo:<br>● No later than when the dog is four months old<br>● No later than four weeks after acquisition/import into Sweden	The dog owner must register their ownership in the national dog register.	No legislation identified.	No legislation identified.	No.
**Norway**	Private organisations can establish and operate a solution for ID and registration<br>The King may order compulsory ID and registration.	Private organisations can establish and operate a solution for ID and registration<br>The King may order compulsory ID and registration.	No legislation identified.	No legislation identified.	No.
**England**	It is compulsory for the dog to carry its ID via a microchip from eight weeks of age at the latest.<br>People who have a license to commercial dog breeding must make sure that puppies are microchipped and registered	There are several databases approved by the authorities.<br>The dog must be registered in one of these.	When in public, the dog must wear a collar stating the name and address of the owner.<br>Exempted from this are dogs who are used for different work purposes.	No legislation identified.	No.

	with the license holder before they are sold.				
**The Netherlands**	No later than when the puppy is seven weeks old.<br><br>It is the breeder's responsibility to get the dog microchipped and registered before sale.	There are several approved databases.<br><br>Puppies must be registered no later than when they are eight weeks old.<br><br>When importing a dog, it must be registered no later than two weeks after arrival.	It is punishable if the dog is in public without a collar or a tag stating information about the owner.	No legislation identified.	Yes, in some municipalities.
**Germany**	<u>Schleswig-Holstein</u><br>Dogs over the age of three months must be microchipped.<br><br><u>Hamburg</u><br>Dogs over the age of three months must be microchipped.<br><br><u>North Rhine-Westphalia</u><br>Only required for dogs for which a permit must be obtained.	<u>Schleswig-Holstein</u><br>No legislation identified.<br><br><u>Hamburg</u><br>A dog must be registered in a dog register from three months of age at the latest, or no later than two weeks after acquisition.<br><br><u>North Rhine-Westphalia</u><br>No legislation identified.	<u>Schleswig-Holstein</u><br>When dogs are in public, they must wear a collar or the like stating the ID and information about the owner.<br><br><u>Hamburg</u><br>No legislation identified.<br><br><u>North Rhine-Westphalia</u><br>No legislation identified.	<u>Schleswig-Holstein</u><br>Compulsory for "dangerous dogs".<br><br>Recommended for "non-dangerous dogs"<br><br><u>Hamburg</u><br>Compulsory for all dogs.<br><br><u>North Rhine-Westphalia</u><br>Compulsory for dog for which a permit must be obtained ("dangerous dogs", dogs of certain breeds and large dogs).	Yes - governed on federal state/municipal level.<br><br><u>Schleswig-Holstein</u><br>In some municipalities.<br><br>A passed "Sachkundeprüfung" can result in reduced dog tax.<br><br><u>Hamburg</u><br>All municipalities, from when the dog is three months old. Higher rates for "dangerous dogs".<br><br><u>North Rhine-Westphalia</u><br>All municipalities. Some municipalities have different rates for difference breeds.
**Austria**	<u>Federal level</u><br>All dogs must carry their ID via a microchip from three months of age at the latest.	<u>Federal level</u><br>National register - the federal ministry is responsible for the database.<br><br>The owner must register the dog or let the veterinarian register the dog no later than one month after acquisition.	No legislation identified.	<u>Vienna</u><br>Yes.<br><br><u>Tirol</u><br>Must be taken out within one month after acquisition.<br><br><u>Vorarlberg</u><br>No legislation identified.	Yes - governed on federal state/municipal level.<br><br><u>Vienna</u><br>Dog tax for dogs that are three months or older. Possibility for reduction and exemption for some dogs.<br><br><u>Tirol</u><br>Dog tax for dogs that are three months or older. The dog must

					be registered within one week after acquisition.<br><br><u>Vorarlberg</u><br>Yes - governed on municipal level. In several municipalities, the rate is higher for “fighting dogs”. In some municipalities, guard dogs, guide dogs and the like are exempted.
**Italy**	The dog must be microchipped from two months of age at the latest.	National dog register governed by the state.<br><br>The dog must be registered at two months of age at the latest.<br><br>Furthermore, regional registers exist and these are correlated with the national register.<br><br>In addition, there are local registers for dogs with a high risk of aggression managed by the local veterinarian (ASL – Azienda sanitaria locale).	No legislation identified.	Only for dogs registered in the register for dogs with high risk of aggression.	Yes.<br><br>Exemptions for certain dogs (assistance dogs, guide dogs etc.).
**The USA**	No microchip requirements.<br><br>Dogs classified as “dangerous” must be microchipped in some states/counties.	No registration requirements.<br><br>Dogs classified as “dangerous” must be registered in some states/counties.<br><br>Microchip registers exist, but they are not correlated.	It is compulsory that the dog can be identified, e.g. by wearing a dog tag.	Liability insurance not required.<br><br>Compulsory dog insurance for dogs classified as “dangerous” in some states/counties.<br><br>Dogs are often included in the home insurance.<br><br>Some insurance companies increase the premium or refuse to insure a dog or the dog owner’s home if the dog is of a certain breed or classified as “dangerous”.	Annual license fee including dog keeping tax required.<br><br>In many states, the license fee is lower for neutered dogs.
**Australia**	<u>New South Wales</u>	<u>New South Wales</u>	<u>New South Wales</u>	<u>New South Wales</u>	<u>New South Wales</u>

	Puppies must be microchipped when they are sold/passed on or at 12 weeks of age at the latest (with a few exemptions).	Puppies must be registered when they are sold/passed on or at 12 weeks of age at the latest.<br><br>Every state has its own register - the 'NSW Pet Registry' in New South Wales.	The dog must wear a dog tag.	No legislation identified.	Lifelong registration fee.<br><br>Annual fee for owners of dogs classified as "restricted" or "dangerous".
**New Zealand**	Microchip required for:<br><br>● All dogs who are registered for the first time after 2006 (except working dogs on farms)<br>● Dogs who are caught/seized for the second time<br>● Unregistered dogs who are caught<br>● Dogs classified as "dangerous" or "menacing"	All dogs over the age of three months must be registered with the local authorities.<br><br>Imported dogs or dogs moved to a new area must be registered there.<br><br>The registration must be renewed on an annual basis.<br><br>Local authorities must provide information to the national dog database.	Upon registration, the dog receives a tag/chip. The dog must wear this at all times.	No legislation identified.	Annual registration fee.<br><br>Local authorities may provide the possibility of a reduced registration fee, for instance for working dogs, owners who have attended a dog owner course etc.<br><br>In several areas, a special permit is required to keep two or three dogs in urban areas.

4.2 Links: ID, registration, insurance and taxes in relation to dog keeping

Denmark:

- Bekendtgørelse af lov om hunde https://www.retsinformation.dk/eli/lta/2021/329
- Bekendtgørelse om mærkning og registrering af hunde https://www.retsinformation.dk/eli/lta/2018/1044

Sweden:

- Lag om tillsyn över hundar och katter https://lagen.nu/2007:1150#P11S1
- Förordning om tillsyn över hundar https://lagen.nu/2007:1240
- Föreskrifter om ändring i Statens jordbruksverks föreskrifter (SJVFS 2008:41) om märkning och registrering av hundar https://djur.jordbruksverket.se/download/18.141c9f5d161fe950f26179a2/1520407115897/2018-003.pdf

Norway:

- Lov om hundehold https://lovdata.no/dokument/NL/lov/2003-07-04-74?q=hundelov

England:

- https://www.gov.uk/get-your-dog-microchipped
- The Microchipping of Dogs (England) Regulations 2015 https://www.legislation.gov.uk/uksi/2015/108/introduction
- The Animal Welfare (Licensing of Activities Involving Animals) (England) Regulations 2018 The Animal Welfare (Licensing of Activities Involving Animals) (England) Regulations 2018 (legislation.gov.uk)
- The Control of Dogs Order 1992 https://www.legislation.gov.uk/uksi/1992/901/contents/made

The Netherlands:

- https://www.rvo.nl/onderwerpen/agrarisch-ondernemen/dieren/dieren-registreren/honden
- Gemeentewet https://wetten.overheid.nl/BWBR0005416/2020-01-01

Germany:

Schleswig-Holstein

- Gesetz über das Halten von Hunden http://www.gesetze-rechtsprechung.sh.juris.de/jportal/?quelle=jlink&query=HuG+SH&psml=bsshoprod.psml&max=true&aiz=true
- https://www.tiere-online.de/hunde/hundesteuer-schleswig-holstein/

Hamburg

- Hundesteuergesetz http://www.landesrecht-hamburg.de/jportal/portal/page/bshaprod.psml?showdoccase=1&doc.id=jlr-HuStGHA1995rahmen&doc.part=X
- Hundesteuer in Hamburg – Kosten und Übersicht (tiere-online.de)

North Rhine-Westphalia

- Hundegesetz für das Land Nordrhein-Westfalen https://recht.nrw.de/lmi/owa/br_bes_text?anw_nr=2&bes_id=5116&gld_nr=2&ugl_nr=2060&menu=1&sg=0&aufgehoben=N&keyword=hund#det
- https://www.tiere-online.de/hunde/hundesteuer-nordrhein-westfalen/

Austria:
Federal level

- Bundesrecht konsolidiert: Gesamte Rechtsvorschrift für Tierschutzgesetz https://www.ris.bka.gv.at/GeltendeFassung.wxe?Abfrage=Bundesnormen&Gesetzesnummer=20003541

Vienna

- Landesrecht konsolidiert Wien: Gesamte Rechtsvorschrift für Hundeabgabegesetz https://www.ris.bka.gv.at/GeltendeFassung.wxe?Abfrage=LrW&Gesetzesnummer=20000125
- https://www.wien.gv.at/finanzen/abgaben/ahs-info/hundesonderregelung.html

Tirol

- Landesrecht konsolidiert Tirol: Gesamte Rechtsvorschrift für Hundesteuergesetz, Tiroler https://www.ris.bka.gv.at/GeltendeFassung.wxe?Abfrage=LrT&Gesetzesnummer=20000184
- Landesrecht konsolidiert Tirol: Gesamte Rechtsvorschrift für Landes-Polizeigesetz https://www.ris.bka.gv.at/GeltendeFassung.wxe?Abfrage=LrT&Gesetzesnummer=20000176
- https://www.tirol.gv.at/fileadmin/themen/gesundheit-vorsorge/veterinaer/downloads/Hundebroschuere_-_Damit_Ihr_Hund_allen_Freude_macht.pdf

Vorarlberg

- https://www.hohenems.at/de/stadt-hohenems/amtsinfo/abgaben_-tarife-und-gebuehren/hundesteuer
- https://www.bregenz.gv.at/buergerservice/dienstleistungen/detail/hundeabgabe/
- https://www.gemeinde-weiler.at/burgerservice/steuern-und-gebuhren/hundesteuer/

Italy:
- Legge 281/1991 http://www.salute.gov.it/imgs/C_17_normativa_911_allegato.pdf
- http://www.salute.gov.it/imgs/C_17_opuscoliPoster_41_allegato.pdf

The USA:
- Personal communication with Andrew Rowan, PhD, and Lisa Moses, VMD, DACVIM

Australia:
New South Wales

- https://legislation.nsw.gov.au/view/html/inforce/current/act-1998-087#sec.12

New Zealand:

- https://www.dia.govt.nz/diawebsite.nsf/wpg_URL/Resource-material-Dog-Control-Dog-Control-Amendment-Act-2003
- https://www.aucklandcouncil.govt.nz/dogs-animals/register-your-dog/Pages/register-dog-first-time.aspx
- https://wellington.govt.nz/dogs-and-other-animals/dogs/register-your-dog/register-your-dog
- https://www.aucklandcouncil.govt.nz/dogs-animals/register-your-dog/Pages/replacement-disc-strap-for-dog.aspx

5. Specific requirements for dog management and physical environment for dog keeping

5.1 Table: Specific requirements for dog management and physical environment for dog keeping

	Requirements for cage/room size for the dog	Limit for time alone	Walking requirements	Ban against tying up dogs for longer periods of time	Car transport requirements	Dog doping forbidden	Painful collars forbidden
**Denmark**	No legislation identified.	No legislation identified.	No legislation identified for private dogs.	It is forbidden to tie up a dog on a permanent basis. When a dog is tied up, the chain must be at least five metres long, and the dog must have access to a kennel of a certain configuration.	Dogs are considered as goods and must, according to the Danish traffic code, be placed so that they do not block the driver's view or hinder the driver from manoeuvring the car.	No legislation identified.	Shock collars and spiked collars are forbidden.<br><br>Martingale collars may be used. These may be furnished with blunt metal foldings or studs no longer than eight mm from the lower edge of the following coupling.
**Sweden**	Minimum measurements defined for all dogs for:<br>● Indoor keeping areas<br>● Runs<br>● Cages, kennels, pet carriers	Dogs must be walked at least every 6th hour. Consequently, it cannot be alone for more than six hours.<br><br>The dog must be gradually accustomed to being alone.	The dog must be walked outside every day.<br><br>Dogs who are kept in a run must be walked at another place every day.	Dogs cannot be tied up, only briefly and supervised, or:<br>● In connection to brief rest<br>● During competitions or hunting<br>● Rest/sleep during travels<br>● In areas where dogs are forbidden<br><br>The dog must be provided with good lying comfort.	The dog must be securely fastened when braking (seat belt or cage).<br><br>If the dog is placed in the boot, it must be supervised.<br><br>Transport cage requirements. However, general minimum measurements can be exempted during transport in connection to travels.<br><br>The dog must not be kept in the vehicle for more than three	Animal doping for competitions or training purposes is forbidden.	Spiked dog collars are forbidden.<br><br>Generally speaking, it is forbidden to use equipment or devices, which give animals an electric shock to affect their behaviour.

					hours when the vehicle is parked.		
**Norway**	No legislation identified.<br>In the guidelines about dogs, requirements are listed regarding:<br>● Indoor rooms<br>● Place to lie<br>● Run	No legislation identified.<br>According to the guidelines about dogs, the dog must not be alone longer than a regular working day.	No legislation identified.<br>According to the guidelines about dogs, the dog must be walked at least three times daily to relieve itself. Furthermore, the dog must be exercised and stimulated daily exercise - for most dogs, it is not sufficient to run loose in the garden.	No legislation identified.<br>In the guidelines about outdoor dog keeping, it is stated that the chain must be at least two metres long, and requirements for stimulation and movement are specified.	The dog is considered as goods, and a car must be securely loaded; this means that the dog must be fastened either in a cage or via a seat belt.<br>According to the guidelines about dogs, the dog must not be in a cage in the car for a longer period of time.	Pertaining to animals in general, the use of means or treatments during training that make the activity unsafe from an animal welfare point of view is forbidden.<br>According to the guidelines about dogs, performance-enhancing treatments are forbidden.	The use of shock collars is forbidden in connection to dog training,<br>Exempted are people who train dogs not to hunt grazing animals, reindeer and cloven-hooved deer, and who meet certain competence requirements.
**England**	Animal owners in general are obliged to ensure a suitable environment for the animal.<br>"Code of practice for the welfare of dogs" provides guidelines for a "suitable" environment.	No legislation identified.<br>"Code of practice for the welfare of dogs" recommends that the dog should only be alone for as long as it feels safe.	No legislation identified.<br>"Code of practice for the welfare of dogs" recommends daily exercise, unless this is contraindicated.	No legislation identified.	Dogs must be securely fastened so that they do not distract the driver or harm themselves or others during braking.<br>"Code of practice for the welfare of dogs" recommends to ensure that the dog is comfortable and safe during transport.	No legislation identified.	No legislation identified.<br>In 2018, it was decided to ban shock collars in England, but the ban has not yet become effective.
**The Netherlands**	Requirements for the layout of runs.	No legislation identified.	No legislation identified.	A dog can only be tied up if:<br>● There is no risk of strangling or injury<br>● The leash has a length that enables the dog to move<br>● The dog has access to a cage that	No legislation identified.	No legislation identified.	It is forbidden to tie up animals with objects which inflict pain on the animals by means of sharp components.<br>A ban against shock collars is planned for 2021.

				protects it from the weather<br><br>It is forbidden to use dogs for dragging, unless it takes place in relation to the sport of dog sledding, and in that case it, only applies to the following breeds:<br>● Alaskan malamute<br>● Eskimo dog<br>● Greenland dog<br>● Samoyed<br>● Siberian husky			
**Germany**	Requirements for the layout of:<br>● Indoor space<br>● Place to lie<br>● Run	No legislation identified.	No legislation identified.<br><br>According to the German Minister for Food and Agriculture, rules for dog walking will be issued in 2021.	It is forbidden to tie up the following:<br>● Dogs below the age of 12 months<br>● Pregnant and nursing bitches<br>● Sick dogs<br><br>Other dogs may be tied up if the following requirements are met:<br>● The leash must be able to run freely along a construction of at least six metres<br>● The dog must be able to move in a range of five metres	Dogs are considered as goods and must be securely fastened in order not to harm people in the car during braking or the like.	It is forbidden to give performance-enhancing drugs to animals in general.	It is forbidden to use electricity to affect the behaviour of an animal, especially their movement.<br><br>It is forbidden to train animals in a way which inflicts significant pain, distress or harm to the animal.

				● The dog must be able to seek its shelter/place to lie<br>● Area, surface and harness/collar must be in such condition that is does not harm the dog<br><br>(Amendments to the law are expected in 2021, making tying up dogs forbidden).			
**Austria**	Requirements for:<br>● Indoor space<br>● Run<br>● Outdoor facilities such as a place with shelter (only for dogs who are suited to be outside)	No legislation identified.	No legislation identified.	Dogs must not be tied up via a chain or the like - not even temporarily. Being kept on a leash is not considered as being tied up.	Dogs are considered as goods and must be securely fastened in order not to harm people in the car during braking or the like.	Doping animals in general is forbidden.	The use of spiked, choke or shock collars are forbidden with animals in general.
**Italy**	The owner is responsible for ensuring regular cleaning of the premises.<br><br>The law cannot forbid animal keeping in flats.	No legislation identified.	The owner is responsible for ensuring adequate physical and mental stimulation of the dog.	No legislation identified.	Transporting an animal cannot hinder or endanger the driving.<br><br>If more animals are transported, they must be kept securely in a cage, behind a net in the boot or the like.	Dog doping is forbidden.	It is forbidden to expose animals to unnecessary distress or force them to a behaviour or work that does not correspond to the features of the animal.<br><br>Shock collars are not directly mentioned, but legal documents from court rulings regarding the use of shock collars and electric anti-bark collars consider both as a criminal offence

							in the form of animal cruelty.
**The USA**	No legislation identified.	No legislation identified.	No legislation identified.	No legislation identified.	A few states have requirements for or forbid transport of dogs/animals on an open truck bed.	No legislation identified.	No legislation identified.
**Australia**	New South Wales<br>No legislation identified.	New South Wales<br>No legislation identified.	New South Wales<br>No legislation identified.	New South Wales<br>It is forbidden to tie up an animal for an unreasonable period of time, with an unreasonably heavy or short device.	New South Wales<br>An animal cannot be transported in a way that inflicts unreasonable, unnecessary or unjustified pain on the animal.<br>Dogs transported on an open truck bed (except for dogs who are used to working with domestic animals) on a public road must be securely fastened/tied or be kept in a cage.	New South Wales<br>No legislation identified.	New South Wales<br>It is forbidden to use electric devices on animals from which the animals cannot escape.
**New Zealand**	The minimum requirement states that the dog must be able to stand erect and lie in a natural position in its place to lie.<br>Best practice provides recommended measurements for kennel and run.	No legislation identified.	Dogs must be exercised on a daily basis. This can take place by means of exercise equipment such as a treadmill. In that case, the exercise must be supervised.<br>Best practice recommends at least 60 minutes of free run per day, taking the dog's age, breed etc. into consideration.	Dogs cannot be tied up if it is harmful or stressful to them. If a dog is tied up without supervision, it must be ensured that the dog cannot be injured.	The dog must be securely fastened during transportation, ventilation must be ensured, and if the dog is left in the car, the driver must make sure that the dog does not show signs of overheating.<br>If a dog is transported on an open truck bed/an open trailer on a public road, the driver must ensure	No legislation identified.	Collars cannot cause wounds, bleeding or swelling or hinder the dog in breathing normally.<br>Spiked collars are forbidden, but electric training devices are not if they are used in a way that do not cause unreasonable or unnecessary pain or di(stress).<br>Best practice recommends to only

					that the dog does not fall of, e.g. by tying it or keeping it in a cage.<br><br>Best practice recommends that the dog is transported in a specially designed transport cage, that the dog is not left alone in a car etc.		use shock collars if other training methods have failed to work.

5.2 Links: Specific requirements for dog management and physical environment for dog keeping

Denmark:

- Bekendtgørelse af lov om hunde https://www.retsinformation.dk/eli/lta/2021/329
- Bekendtgørelse om dyrevelfærdsmæssige mindstekrav til hold af hunde https://www.retsinformation.dk/eli/lta/2020/1749
- Bekendtgørelse af færdselsloven https://www.retsinformation.dk/eli/lta/2018/1324

Sverige:

- Statens jordbruksverks föreskrifter och allmänna råd om hållande av hundar och katter https://www.hsb.se/contentassets/9b52bd940f684adab136bbc99f7b42f2/kalla-foreskrift-om-nya-regler-for-hundar-och-katter-sjvfs2020-8.pdf
- Föreskrifter och allmänna råd om träning och tävling med djur https://lagen.nu/sjvfs/2019:26
- Djurskyddsförordning https://lagen.nu/2019:66

Norway:

- Veileder for hold av hund utendørs https://www.mattilsynet.no/om_mattilsynet/gjeldende_regelverk/veiledere/veileder_for_hold_av_hund_utendors.3825/binary/Veileder%20for%20hold%20av%20hund%20utend%C3%B8rs
- Tilsynsveileder hund Tilsynsveileder - hund (mattilsynet.no)
- Lov om vegtrafikk https://lovdata.no/dokument/NL/lov/1965-06-18-4?q=last%20kj%C3%B8ret%C3%B8y
- https://www.mattilsynet.no/dyr_og_dyrehold/kjaledyr_og_konkurransedyr/hund/hunden_paa_biltur.16358
- Forskrift om bruk av elektrisk strøm ved trening av hund https://lovdata.no/dokument/SF/forskrift/2008-03-14-256?q=forskrift%20om%20bruk%20av%20elektrisk

England:

- Animal Welfare Act 2006 https://www.legislation.gov.uk/ukpga/2006/45/contents
- Code of practice for the welfare of dogs https://assets.publishing.service.gov.uk/government/uploads/system/uploads/attachment_data/file/697953/pb13333-cop-dogs-091204.pdf
- https://www.gov.uk/government/news/cruel-electric-shock-collars-for-pets-to-be-banned--2

Nederlandene:

- Regeling houders van dieren https://wetten.overheid.nl/BWBR0035248/2020-01-16#Hoofdstuk8
- Besluit houders van dieren https://wetten.overheid.nl/BWBR0035217/2018-07-01

Germany:

- Tierschutzgesetz http://www.gesetze-im-internet.de/tierschg/BJNR012770972.html
- Tierschutz-Hundeverordnung http://www.gesetze-im-internet.de/tierschhuv/__5.html
- Strassenverkehrs-Ordnung http://www.gesetze-im-internet.de/stvo_2013/__22.html
- https://rp-online.de/politik/deutschland/pflicht-fuer-hundehalter-neue-verordnung-zweimal-taeglich-gassigehen_aid-52789945

Austria:

- Bundesrecht konsolidiert: Gesamte Rechtsvorschrift für Tierschutzgesetz https://www.ris.bka.gv.at/GeltendeFassung.wxe?Abfrage=Bundesnormen&Gesetzesnummer=20003541
- Bundesrecht konsolidiert: Gesamte Rechtsvorschrift für 2. Tierhaltungsverordnung https://www.ris.bka.gv.at/GeltendeFassung.wxe?Abfrage=Bundesnormen&Gesetzesnummer=20003860
- Kraftfahrgesetz §101 https://www.ris.bka.gv.at/GeltendeFassung.wxe?Abfrage=Bundesnormen&Gesetzesnummer=10011384
- Landesrecht konsolidiert Tirol: Gesamte Rechtsvorschrift für Landes-Polizeigesetz https://www.ris.bka.gv.at/GeltendeFassung.wxe?Abfrage=LrT&Gesetzesnummer=20000176

Italy:

- https://www.oipa.org/italia/leggi/CONSIGLI/il%20cane%20e%20la%20legge.pdf
- Ordinanza 3 marzo 2009 http://www.salute.gov.it/imgs/C_17_normativa_1915_allegato.pdf
- http://www.salute.gov.it/imgs/C_17_opuscoliPoster_41_allegato.pdf
- Articolo 169 codice della strada https://www.brocardi.it/codice-della-strada/titolo-v/art169.html
- Legge 189/2004 https://www.lav.it/cpanelav/js/ckeditor/kcfinder/upload/files/files/Legge%20189-2004.pdf
- https://www.litis.it/2013/09/26/il-collare-antiabbaio-e-strumento-di-maltrattamento-dei-cani-cassazione-380342013/
- https://www.animal-law.it/rivista-diritti-animali/diritto/collare-elettrico-e-lecito-utilizzarlo/

The USA:

- Personal communication with Andrew Rowan, PhD, and Lisa Moses, VMD, DACVIM
- https://www.animallaw.info/article/faq-dogs-transported-pickup-truck-beds

Australia:

New South Wales

- https://legislation.nsw.gov.au/view/whole/html/inforce/current/act-1979-200#sec.7
- https://legislation.nsw.gov.au/view/whole/html/inforce/current/sl-2012-0408#sch.3
- https://legislation.nsw.gov.au/view/whole/html/inforce/current/act-1979-200#sec.10

New Zealand:

- https://www.mpi.govt.nz/dmsdocument/1407-Transport-within-New-Zealand-Animal-Welfare-Code-of-Welfare
- Code of welfare – dogs https://www.mpi.govt.nz/dmsdocument/1428-Dogs-Animal-Welfare-Codes-of-Welfare

6. The dog in the public

6.1 Table: The dog in the public

	Leaving a tied up dog forbidden	Leash requirement in cities	Muzzle requirement in cities
**Denmark**	No legislation identified.	In cities and urban areas, the dog must be kept on a leash or be accompanied by a person with full control over the dog.	No general requirements.<br>Dogs that have shown dangerous behaviour may be ordered to wear a muzzle.
**Sweden**	It is allowed to briefly tie up the dog.	Dogs must be kept in a way, which prevents it from causing harm or significant inconvenience.<br>No leash requirement in cities. There may be local bans against bringing dogs to playgrounds, bathing areas etc.	No legislation identified.
**Norway**	It is forbidden to leave a dog tied at the entrance of a building that is open to the public.	A dog may be walked without a leash, if it is accompanied and controlled by the owner.<br>If the dog rushes towards, jumps up, follows or gets in the way of people, it must be kept on a leash in places with common coming and going.<br>The dog owner must be particularly cautious around children.<br>No leash requirement in cities. However, municipalities can establish requirements for using a leash in particular areas like shopping areas, schools, playgrounds etc.	No general requirements.<br>Muzzle order for dogs who pose a danger or instil fear.
**England**	No legislation identified.	No general requirements.<br>The owner is responsible for ensuring that the dog does not get "dangerously out of control".<br>Dogs included in the "index of exempted dogs" must be kept on a leash in public.<br>Owners of dogs that have been "dangerously out of control" may be required to keep the dog on a leash.	No general requirements.<br>Dogs included in the "index of exempted dogs" must wear a muzzle in public.<br>Dogs that have been "dangerously out of control" may be required to wear a muzzle.

**The Netherlands**	No legislation identified.	It is forbidden to let the dog walk around in an urban area without a leash.	No legislation identified.
**Germany**	No legislation identified.	<u>Schleswig-Holstein</u><br>All dogs must be kept on a leash in places with many people (pedestrian streets, parks, public buildings, sports facilities, cemeteries, markets etc.).<br><br>All dogs classified as “dangerous” must be kept on a leash in public.<br><br><u>Hamburg</u><br>All dogs in Hamburg must be kept on a leash in public.<br><br>A leash length order of maximum two metres applies to:<br><br>● Dogs that have repeatedly bothered people or animals<br>● Bitches in heat<br>● Dogs brought to places with many people<br>● Dogs brought to areas near schools, playgrounds and other places with children/young people<br><br>It is possible to be exempted from this order by documenting a passed obedience test (Gehörsamsprüfung). However, this only applies to non-dangerous dogs and only in some areas.<br><br><u>North Rhine-Westphalia</u><br>Dogs must be kept on a leashsh in areas with many people (shopping areas, parks, playgrounds, markets, schools etc.).<br><br>Dogs who require a permit must always be kept on a leash.	<u>Schleswig-Holstein</u><br>Only dogs over the age of six months who are classified as “dangerous”.<br><br><u>Hamburg</u><br>Only dogs over the age of nine months who are classified as “dangerous”. Some “dangerous” dogs may be exempted from the muzzle order after a test.<br><br><u>North Rhine-Westphalia</u><br>Only dogs over the age of six months who are classified as “dangerous”.
**Austria**	No legislation identified.	<u>Vienna</u><br>Dogs must be kept on a leash and wear a muzzle in public. Dogs must always be kept on a leash in public parks and marked green areas.<br><br><u>Tirol</u><br>Dogs must be kept on a leash or wear a muzzle in a range of public places such as large gatherings, public transportation, childcare areas etc.	<u>Vienna</u><br>The dog must wear a muzzle or be kept on a leash in public. The dog must wear a muzzle in public places with many people (public transportation, shops etc.).<br><br><u>Tirol</u><br>Dogs must be kept on a leash or wear a muzzle in a range of public places such as large gatherings, public transportation, childcare areas etc.

		Vorarlberg<br>The rules in this area are established on municipality level.	Vorarlberg<br>The rules in this area are established on municipality level.
**Italy**	No legislation identified.	The dog must be kept on a leash with a maximum length of 1.5 metres in urban areas and places open to the public.	The owner must always bring a muzzle when in public in order to place it on the dog if it poses a risk to others or upon request from the authorities.
**The USA**	No legislation identified.	Many states require that the dogs is kept on a leash or is controlled in another way when in public.<br>Dogs running loose without the owner nearby and without a license dog tag may be caught by 'dog control officers'.	Only applicable to dogs who are classified as "dangerous".
**Australia**	New South Wales<br>No ban against placing the dog in front of a shop, but it is forbidden to tie up a dog for an unreasonable period of time.	New South Wales<br>Dogs must be kept on a leash and be accompanied by a person with control over the dog. It is considered "not under control" to have more than four dogs with you.<br>Exemptions from this is for instance dogs at work, dogs at competitions, or areas in which dogs may run free etc.<br>There may be areas where dogs are not allowed, e.g. playgrounds, schools etc.	New South Wales<br>Only applicable to dogs classified as "restricted", "dangerous" or "menacing".
**New Zealand**	No legislation identified.	Dogs must be kept on a leash, or the owner must bring a leash, when in public.<br>Local authorities can:<br>● Forbid dogs from accessing certain public places<br>● Order the dog to be kept on a leash in certain public places<br>● Establish minimum requirements for dog keeping (e.g. limit to the number of dogs per property, requirement to keep dogs tied up or enclosed in certain time periods, keep bitches in oestrous enclosed)<br>Best practice recommends that dogs are under constant control or enclosed during exercise in order for them not to harm anyone.	Only applicable to dogs classified as "dangerous" or "menacing".

6.2 Links: The dog in the public

Denmark:

- Bekendtgørelse af lov om hunde https://www.retsinformation.dk/eli/lta/2021/329

Sweden:

- Lag om tillsyn över hundar och katter https://lagen.nu/2007:1150
- Statens jordbruksverks föreskrifter och allmänna råd om hållande av hundar och katter https://www.hsb.se/contentassets/9b52bd940f684adab136bbc99f7b42f2/kalla-foreskrift-om-nya-regler-for-hundar-och-katter-sjvfs2020-8.pdf

Norway:

- Lov om hundehold https://lovdata.no/dokument/NL/lov/2003-07-04-74?q=lov%20om%20hunder
- Båndtvang - Norsk Kennel Klub (nkk.no)

England:

- Dangerous Dogs Act 1991 https://www.legislation.gov.uk/ukpga/1991/65/introduction#commentary-c932828

The Netherlands:

- Besluit OM-afdoening https://wetten.overheid.nl/BWBR0022233/2020-01-01#BijlageI

Germany:

Schleswig-Holstein

- Gesetz über das Halten von Hunden http://www.gesetze-rechtsprechung.sh.juris.de/jportal/?quelle=jlink&query=HuG+SH&psml=bsshoprod.psml&max=true&aiz=true

Hamburg

- Hamburgisches Gesetz über das Halten und Führen von Hunden http://www.landesrecht-hamburg.de/jportal/portal/page/bshaprod.psml?showdoccase=1&st=lr&doc.id=jlr-HuGHArahmen&doc.part=X&doc.origin=bs

North Rhine-Westphalia

- Hundegesetz für das Land Nordrhein-Westfalen https://recht.nrw.de/lmi/owa/br_bes_text?anw_nr=2&bes_id=5116&gld_nr=2&ugl_nr=2060&menu=1&sg=0&aufgehoben=N&keyword=hund#det

Austria:

Vienna

- Landesrecht konsolidiert Wien: Gesamte Rechtsvorschrift für Wiener Tierhaltegesetz https://www.ris.bka.gv.at/GeltendeFassung.wxe?Abfrage=LrW&Gesetzesnummer=20000404

- https://www.wien.gv.at/gesellschaft/tiere/haustiere/hunde/

Tirol

- Landesrecht konsolidiert Tirol: Gesamte Rechtsvorschrift für Landes-Polizeigesetz https://www.ris.bka.gv.at/GeltendeFassung.wxe?Abfrage=LrT&Gesetzesnummer=20000176

Vorarlberg

- https://vorarlberg.at/web/land-vorarlberg/contentdetailseite/-/asset_publisher/qA6AJ38txu0k/content/hunde-haltung-hundekauf-kennzeichnung-und-registrierung?article_id=408853

Italy:

- http://www.salute.gov.it/imgs/C_17_opuscoliPoster_41_allegato.pdf

The USA:

- Personal communication with Andrew Rowan, PhD, and Lisa Moses, VMD, DACVIM
- https://www.animallaw.info/article/detailed-discussion-state-dog-impound-laws
- https://www.animallaw.info/article/frequently-asked-questions-local-dog-laws
- https://www.animallaw.info/article/designing-model-dog-park-law

Australia:

New South Wales

- https://legislation.nsw.gov.au/view/whole/html/inforce/current/act-1979-200#sec.10
- https://www.olg.nsw.gov.au/public/dogs-cats/responsible-pet-ownership/faqs/
- https://www.olg.nsw.gov.au/public/dogs-cats/responsible-pet-ownership/declared-dangerous-and-menacing-dogs/
- https://www.olg.nsw.gov.au/public/dogs-cats/responsible-pet-ownership/restricted-dogs/
- https://legislation.nsw.gov.au/view/html/inforce/current/act-1998-087#sec.14

New Zealand:

- Dog control act https://legislation.govt.nz/act/public/1996/0013/latest/whole.html?search=ts_act%40bill%40regulation%40deemedreg_dog_resel_25_a&p=1#DLM374489

7. Surgery and killing

7.1 Table: Surgery and killing

	Neutering	Vaccine requirement (not related to travelling)	Tail docking forbidden	Ear cropping forbidden	Removal of vocal cords forbidden	Limitations regarding killing of dogs
**Denmark**	Neutering dogs is allowed without a veterinary cause.<br>Only veterinarians are allowed to perform a dog castration. Anaesthesia and subsequent pain treatment are required	No legislation identified.	Tail docking a dog is forbidden.<br>Puppies of the following breeds can be tail docked if the procedure is performed by a veterinarian with anaesthesia and subsequent pain treatment before the puppy is four days old:<br>● Wirehaired pointer<br>● Shorthaired pointer<br>● Weimaraner<br>● Vizsla<br>● Brittany	Ear cropping a dog is forbidden.	Removal of a dog's vocal cords is forbidden.	No limitations to when a dog can be euthanised.<br>Veterinarians, butchers, people with a hunting licence or people who are educated within slaughtering and killing are allowed to euthanise dogs.<br>Puppies who are less than one week old can be euthanised by everyone (except children below the age of 15 years).<br>There are requirements for the killing method of dogs.
**Sweden**	Neutering dogs is generally allowed without a veterinary cause.<br>Surgery on animals must be performed by a veterinarian.	No legislation identified.	Generally, it is forbidden to perform surgery on animals without a veterinary cause.	Generally, it is forbidden to perform surgery on animals without a veterinary cause.	Generally, it is forbidden to perform surgery on animals without a veterinary cause.	No limitations to when a dog can be euthanised.<br>There are requirements for the killing method of dogs.
**Norway**	Neutering a dog without a veterinary cause is forbidden.	No legislation identified.	Generally, it is forbidden to perform surgery on animals without a veterinary cause.	Generally, it is forbidden to perform surgery on animals without a veterinary cause.	Generally, it is forbidden to perform surgery on animals without a veterinary cause.	No limitations to when a dog can be euthanised.

						There are requirements for the killing method of dogs.
**England**	No legislation identified.	No legislation identified.<br>"Code of practice for the welfare of dogs" recommends following vaccine programmes.	Tail docking a dog is forbidden,<br>except for certified working dogs as well as the following dog types:<br>● Breeds of hunt/point/retrieve types<br>● Spaniels<br>● Terriers	Pertaining to animals in general, it is forbidden to perform surgery affecting sensitive tissue or bone, unless it has a veterinary cause.	Pertaining to animals in general, it is forbidden to perform surgery affecting sensitive tissue or bone, unless it has a veterinary cause.	No limitations to when a dog can be euthanised.<br>No requirements for the person who euthanises companion dogs, but it must be carried out humanely.<br>Animals kept in a way that requires a license (e.g. breeding, sale etc.) can only be euthanised by a veterinarian (or a person authorised to do so by a veterinarian).
**The Netherlands**	No legislation identified.	No legislation identified.	Pertaining to animals in general, it is forbidden to perform physical surgeries that do not have a veterinary cause.	Pertaining to animals in general, it is forbidden to perform physical surgeries that do not have a veterinary cause.	Pertaining to animals in general, it is forbidden to perform physical surgeries that do not have a veterinary cause.	It is forbidden to euthanise dogs, except:<br>● To prevent the dog from posing a danger to people or other animals<br>● If a veterinarian has determined that it is in the best interest of the animal<br>● It is done to end distress<br>● It is done because of dangerous behaviour that cannot be corrected.<br><br>Only a veterinarian can euthanise dogs, unless it is an emergency.
**Germany**	Neutering to prevention of reproduction is	No legislation identified.	Complete or partial removal of body parts	Complete or partial removal of body parts	Complete or partial removal of body parts	It is forbidden to euthanise vertebrates

	allowed and must be performed by a veterinarian.		without a veterinary cause is forbidden.<br><br>Hunting dogs can be exempted from this.	without a veterinary cause is forbidden.	without a veterinary cause is forbidden.	without a reasonable cause.<br><br>Only a person educated to do so can euthanise animals.
**Austria**	It is forbidden to perform surgery on animals without a veterinary cause.<br><br>Exempted is neutering to prevent reproduction.	No legislation identified.	Tail docking animals is forbidden.	Ear cropping animals is forbidden.	Removal of animal vocal cords is forbidden.	It is forbidden to euthanise all animals if there is no reasonable cause to do so.<br><br>Only veterinarians can euthanise animals.
**Italy**	The local veterinarian is responsible for neutering stray dogs as a part of the plan to control the dog population.	No legislation identified.	Tail docking a dog is forbidden.	Ear cropping a dog is forbidden.	Removal of a dog's vocal cords is forbidden.	It is forbidden to euthanise animals without a legally acceptable reason.<br><br>Only veterinarians can euthanise dogs and only it the animal is incurably ill or proven dangerous.
**The USA**	No legislation identified.<br><br>License fee reduction for neutered dogs in many states.<br><br>Around 98 % of dogs in the USA are neutered.	No legislation identified.	Tail docking a dog is allowed.	Ear cropping a dog is allowed.	Removal of the vocal cords is allowed in most states.	No limitations to when a dog can be euthanised.<br><br>Requirements for who can euthanise a dog varies from state to state. Some states have rules while others do not.<br><br>Typically, veterinarians and trained animal shelter staff can euthanise dogs.<br><br>If you hold a firearms license, you can shoot a dog in an emergency (if it is suffering or if it attacks domestic animals).
**Australia**	New South Wales	New South Wales	New South Wales	New South Wales	New South Wales	New South Wales

	No legislation identified.<br><br>Registration fee reduction if the dog is neutered.	No legislation identified.	Tail docking a dog is forbidden.	Ear cropping a dog is forbidden.	Removing the vocal cords of a dog (debarking) is forbidden.<br><br>However, if the owner has been required to stop the dog's bothersome barking, and behavioural treatment has been tried and the only remaining solution is killing, a veterinarian can perform this surgery.	No limitations to when a dog can be euthanised.<br><br>It is forbidden to cause an animal to die unreasonably, unnecessarily or unjustifiably.<br><br>As for breeders, killing of dogs must be performed by a veterinarian or another trained person.
**New Zealand**	No legislation identified.<br><br>Best practice recommends that dogs who are not used for breeding should be neutered.	No minimum requirements.<br><br>Best practice recommends that dogs should be vaccinated according to the recommendation of the veterinarian.	Tail docking a dog is forbidden.	Ear cropping a dog is forbidden.	Debarking is not forbidden, but it must be performed by a veterinarian, and only if other attempts at treating the barking behaviour have been unsuccessful.	No limitations to when a dog can be euthanised.<br><br>Killing must be carried out in a way in which death occurs fast and with minimal pain and stress.<br><br>No dogs can be euthanised by means of drowning.<br><br>Best practice recommends that killing is carried out by a veterinarian or another qualified person if no veterinarian is available.

7.2 Links: Surgery and killing

Denmark

- Bekendtgørelse om dyrevelfærdsmæssige mindstekrav til hold af hunde https://www.retsinformation.dk/eli/lta/2020/1749
- Lov om dyrevelfærd https://www.retsinformation.dk/eli/lta/2020/133

Sweden:

- Djurskyddsförordning https://lagen.nu/2019:66
- Djurskyddslag https://lagen.nu/2018:1192
- Förskrifter och allmänna råd om slakt och annan avlivning av djur https://djur.jordbruksverket.se/download/18.7c1e1fce169bee5214fad439/1553851388110/2019-008.pdf
- https://djur.jordbruksverket.se/amnesomraden/djur/olikaslagsdjur/hundarochkatter/skotselavhundochkatt/kuperingtatueringavlivningochandraingrepp.4.207049b811dd8a513dc80001997.html

Norway:

- Forskrift om avliving av hund og katt https://lovdata.no/dokument/SF/forskrift/1998-10-11-991
- Lov om dyrevelferd https://lovdata.no/dokument/NL/lov/2009-06-19-97
- https://www.mattilsynet.no/dyr_og_dyrehold/kjaledyr_og_konkurransedyr/hund/kastrering_av_hund__er_det_tillatt.13955

England:

- Animal Welfare Act 2006 https://www.legislation.gov.uk/ukpga/2006/45/contents
- The Docking of Working Dogs' Tails (England) Regulations 2007 https://www.legislation.gov.uk/uksi/2007/1120/schedule/1
- The Animal Welfare (Licensing of Activities Involving Animals) (England) Regulations 2018 The Animal Welfare (Licensing of Activities Involving Animals) (England) Regulations 2018 (legislation.gov.uk)
- Code of practice for the welfare of dogs https://assets.publishing.service.gov.uk/government/uploads/system/uploads/attachment_data/file/697953/pb13333-cop-dogs-091204.pdf
- https://www.rcvs.org.uk/setting-standards/advice-and-guidance/code-of-professional-conduct-for-veterinary-surgeons/supporting-guidance/killing-of-animals/

The Netherlands:

- Wet dieren https://wetten.overheid.nl/BWBR0030250/2020-01-01/#Hoofdstuk2_Paragraaf1_Artikel2.2
- Regeling houders van dieren https://wetten.overheid.nl/BWBR0035248/2020-01-16#Hoofdstuk5
- https://www.nvwa.nl/onderwerpen/honden-en-katten/welzijnseisen-honden-en-katten#:~:text=Alleen%20dierenartsen%20mogen%20honden%20en%20katten%20doden.%20Dat,een%20ongeluk%20heeft%20gehad%20en%20ernstig%20gewond%20is.

Germany:

- Tierschutzgesetz http://www.gesetze-im-internet.de/tierschg/BJNR012770972.html

Austria:

- Bundesrecht konsolidiert: Gesamte Rechtsvorschrift für Tierschutzgesetz https://www.ris.bka.gv.at/GeltendeFassung.wxe?Abfrage=Bundesnormen&Gesetzesnummer=20003541
- Bundesrecht konsolidiert: Gesamte Rechtsvorschrift für 2. Tierhaltungsverordnung https://www.ris.bka.gv.at/GeltendeFassung.wxe?Abfrage=Bundesnormen&Gesetzesnummer=20003860

Italy:

- Legge 189/2004 https://www.lav.it/cpanelav/js/ckeditor/kcfinder/upload/files/files/Legge%20189-2004.pdf
- Legge 281/1991 http://www.salute.gov.it/imgs/C_17_normativa_911_allegato.pdf
- http://www.salute.gov.it/imgs/C_17_opuscoliPoster_41_allegato.pdf

The USA:

- Personal communication with Andrew Rowan, PhD, and Lisa Moses, VMD, DACVIM
- https://www.animallaw.info/article/brief-summary-animal-euthanasia

Australia:

New South Wales

- https://legislation.nsw.gov.au/view/whole/html/inforce/current/act-1979-200#sec.12
- https://legislation.nsw.gov.au/view/whole/html/inforce/current/sl-2012-0408#sec.20
- https://www.dpi.nsw.gov.au/animals-and-livestock/animal-welfare/general/codes-of-practice/breeding-dogs-and-cats

New Zealand:

- Code of welfare – dogs https://www.mpi.govt.nz/dmsdocument/1428-Dogs-Animal-Welfare-Codes-of-Welfare

8. Import

8.1. Table: Import

	EU rules for import* from third countries as well as trade between EU countries**	Puppies can be exempted from the rabies vaccine requirement in connection to trade within the EU	Other requirements than the mentioned EU rules for trade and import	Ban against import of certain breeds
**Denmark**	Yes.	Yes, in connection to trade with EU countries.<br>However, transit countries around Denmark do not have this exemption. Hence, import via plane may be the only option.<br>The exemption for young animals never applies to import from third countries.	A person importing dogs into Denmark, by means of either trade or import, must be registered with and have a license issued by the authorities.<br>Dogs imported into Denmark, by means of either trade or import, must be registered in the Danish Dog Register no later than four weeks after import.	Same breeds as on the list of banned breeds.<br>The ban applies to both trade and import. Dogs of breeds included on the ban list are allowed to stay briefly in Denmark in connection to transit.
**Sweden**	Yes.	No. Neither in connection to trade nor to import.<br>However, there is no vaccination requirement for dogs from Norway and thus no lower age limit for importing puppies from Norway.	Dogs imported into Sweden in connection to trade within the EU must be imported at a custom house and registered there.<br>Dogs imported into Sweden, by means of either trade or import, must be registered in the central dog register no later than four weeks after import.	No legislation identified.
**Norway**	Regarding animals in general, Norway has requirements for trade with EU countries as well as for import from third countries similar to the EU rules.	No. Neither in connection to trade nor to import.<br>However, there is no vaccination requirement for dogs from Sweden and thus no lower age limit for importing puppies from Sweden.	Stray dogs cannot be imported into Norway for commercial purposes. If the owner wants to import a “street dog” from abroad, they must be able to document that they have been living together for at least six months.<br>A person importing dogs into Norway, by means of both trade and import, must be registered with and have a license issued by the authorities.	It is forbidden to import breeds included in the list of banned breeds.
**England**	The following requirements apply to import:<br>● The establishment, from which the dog derives, must be registered	No. Neither in connection to trade nor to import.	Dogs can only be imported at places with border control and must be examined by a veterinarian.	It is forbidden to import breeds included in the list of banned breeds. The ban applies to both trade and import.

	● Microchip<br>● Rabies vaccine (including an antibody test for non-list third countries)<br>● Treatment for *Echinococcus multilocularis*<br>● Clinical examination no later than 48 hours before import<br>● Health certificate (IPAFFS over TRACES)		APHA (Animal and Plant Health Agency) must be informed through IPAFFS (Import of Products, Animals, Food and Feed System) no later than one day before arrival.<br>The carrier must apply for authorisation to transport live animals.	
**The Netherlands**	Yes.	No. Neither in connection to trade nor to import.	The dog must be registered in an approved database no later than two weeks after import.<br>Requirements for transports longer than 65 km and transports longer than eight hours.	No legislation identified.
**Germany**	Yes.	No. Neither in connection to trade nor to import.	No other requirements identified.	It is forbidden to import dogs of the following breeds or cross-breeds including these breeds:<br>● Pitbull terrier<br>● American staffordshire terrier<br>● Staffordshire bull terrier<br>● Bull terrier
**Austria**	Yes.	Yes, in connection to trade between EU countries.<br>The exemption for young animals never applies to import from third countries.	The dog must be registered by the veterinarian after import.	No legislation identified.
**Italy**	Yes.	No. Neither in connection to trade nor to import.	No other requirements identified.	No legislation identified.
**The USA**	● The dog must appear healthy<br>● The dog must have a valid rabies vaccination (depending on the country from which it is imported)<br>● Valid vaccination for canine distemper virus, hepatitis, leptospirosis, parvovirus, canine parainfluenza virus<br>● The dog must be at least six months old.<br>● No sign of tapeworm infection<br>● Veterinary examination to rule out screwworm (if imported from countries where it exists) five days prior to departure<br>● USDA (US Department of Agriculture) APHIS (Animal and Plant Health Inspection Service) certificate issued by an authorised veterinarian			No bans.

	● Import permission from APHIS<br><br>Furthermore, additional requirements may apply in the individual states.	
**Australia**	<u>Australia in general</u><br>Import from country in Category 1 (New Zealand among others)<br>● The dog must have a microchip<br>● The dog must be at least eight weeks old<br>● Bitches cannot be more than 40 days pregnant<br>● The dog must have spent its entire life or at least 90 days before import in New Zealand<br>● The dog cannot be in quarantine<br>● Health certificate issued by a veterinarian<br>● Health examination five days before departure<br>● Declaration that the country is free from rabies, *Brucella canis*, *Leptospira canicola*, *Ehrlichia canis*, *Leishmania infantum*<br>● If the dog has been to Africa, it must be treated for *Babesia canis* prior to import<br>● Import permit not necessary, but the arrival must be reported to the authorities at least three days before arrival<br>● No quarantine, but the dog must pass through border control<br><br>Import from country in Category 2 (countries declared free from rabies)<br>● Same requirements as for Category 1. Furthermore:<br>● Import permit<br>● Quarantine in an approved quarantine facility for at least 10 days after arrival<br>● Must have lived in the export country since birth or at least 180 days immediately before departure<br>● Bitches cannot be more than 30 days pregnant<br>● Requirements for transportation, transit, in connection to the journey<br>● Tick/flea treatment at least 21 days before blood samples for various infections<br>● Test for *Ehrlichia canis*, *Brucella canis*, *Leishmania infantum*, *Leptospira canicola* (alternatively vaccination), *Babesia canis* (if the dog has been to Africa) 45 (28 days for *Babesia*) days prior to departure<br>● Treatment for internal parasites two times prior to departure<br><br>Import from country in Category 3 (countries without rabies or with controlled rabies)<br>● Same requirements as for Category 2. Furthermore:<br>● The dog must have a valid rabies vaccine, given when the dog is at least 90 days old, and a positive antibody test no older than 24 months must be provided<br>● The dog cannot be imported until 180 days after a positive antibody test<br><br>Import from countries which do not belong in Categories 1, 2 or 3<br>● Special application required<br>● Direct import from Malaysia is not allowed	<u>Australia in general</u><br>It is forbidden to import dogs of the following breeds/types:<br>● Dogo argentino<br>● Fila brasileiro<br>● Japanese tosa<br>● Pitbull terrier or American pitbull<br>● Presa canario<br>● Czechoslovakian wolfdog<br>● Saarloos wolfdog<br>● Lupo italiano<br>● Kunming wolfdog

**New Zealand**	Import from country in Category 1 (Australia)<br>● The dog must have a microchip, and the microchip number must appear in all documents<br>● The dog must be at least eight weeks old and weaned<br>● The dog cannot be more than 42 days pregnant<br>● The dog cannot be of a banned breed<br>● Test (for dogs over the age of six months) and treatment for heartworm<br>● *Babesia canis* (for dogs who have been to South Africa) and *Babesia gibsoni.* A positive *Babesia gibsoni* test eliminates import into New Zealand<br>● Treatment for ectoparasites (lice and fleas) two times prior to departure<br>● Treatment for roundworm and tapeworm two times prior to departure<br>● Final veterinary examination in the two days prior to departure, including examination for canine transmissible venereal tumour<br>● A practising veterinarian can carry out tests and examinations and fill in 'Model Certificate A'<br>● An official veterinarian fills in 'Model Veterinary Certificate B'<br>● The Ministry for Primary Industries (MPI) must be notified five days prior to arrival in New Zealand. MPI issues an import permit<br>● The dog is examined at one of two border control posts<br>● The dog must be registered with the local authorities after arrival<br><br>Import from country in Category 2 (countries declared free from rabies)<br>● Same requirements as for Category 1. Furthermore:<br>● The dog must be at least 12 weeks old<br>● Immediately before departure, the dog must have lived at least six months (or since birth) in the country from which it is imported<br>● Besides the conditions mentioned in Category 1 countries, the dog must be tested or treated for leptospirosis and tested for *Brucella canis* (a positive test eliminates import into New Zealand)<br>● In addition to filling in 'Model Certificate A', the practising veterinarian must fill in the 'Official Veterinarian Declaration'<br>● Upon arrival, the dog must be brought directly to a quarantine facility where it must be kept in quarantine for at least 10 days<br>● The dog must be vaccinated against diseases as required by the quarantine facility, e.g. distemper, hepatitis, kennel cough etc.<br>● The dog must be kept in quarantine in an approved quarantine facility for at least 10 days after arrival<br><br>Import from country in Category 3 (countries without rabies or with controlled rabies)<br>● Same requirements as for Category 2. Furthermore:<br>● The dog must be at least nine months old<br>● The dog must be vaccinated against rabies and have undergone antibody testing 24 months before departure at the earliest and no later than three months before departure	It is forbidden to import dogs of the following breeds/types or cross-breeds where these breeds/types are dominant:<br>● Fila brasileiro<br>● Dogo argentino<br>● Japanese tosa/tosa inu<br>● Presa canario<br>● American pitbull terrier<br><br>Furthermore, it is not possible to import wolf hybrids.

* Here, import covers commercial dog import from third countries (list or non-list countries). However, commercial import of dogs from EU to EU countries (trade) is also covered.

** For all EU countries, the following requirements apply to trade between EU countries: 1) The company who acts as sender must be registered with the authorities. 2) The dog must be microchipped (it can also be tattooed if the tattoo was made before July 2011). 3) Rabies vaccination at the age of 12 weeks at the earliest and completed vaccine schedule 21 days prior to import at the earliest. 4) Treatment for *Echinococcus multilocularis* (tapeworm) carried out by a veterinarian at least 24 hours and no more than 120 hours before import. 5) Clinical examination of the dog carried out by an authorised veterinarian within 48 hours before departure. 6) EU pet passport 7) Health certificate (the TRACES system).

In addition to the requirements for trade between EU countries, the following requirements apply to all EU countries in connection to import from third countries (list countries): 1) Same requirements as for trade within the EU. 2) The import can only take place from approved countries. 3) Import at selected border control posts. 4) Filling in the 'common health entry document (CHED)' at the border control. In addition, with regards to import from third countries (non-list countries), the following applies: Rabies antibody test carried out by an authorised veterinarian at least 30 days after vaccination and at least three months before departure

Transportation of dogs in connection to economic activity (trade and import) must meet the requirements of the Council Regulation (EC) No 1/2005 about animal welfare during transport.

8.2. Links: Import

Denmark:

- https://www.foedevarestyrelsen.dk/english/ImportExport/Commercial_trade-import_of_pet_animals/Pages/Dogs_cats.aspx
- Bekendtgørelse om mærkning og registrering af hunde https://www.retsinformation.dk/eli/lta/2018/1044
- Directive 92/65/EEC (europa.eu) https://eur-lex.europa.eu/legal-content/DA/TXT/PDF/?uri=CELEX:01992L0065-20141229&from=EN
- Regulation (EU) 1/2005 (europa.eu) https://www.europarl.europa.eu/RegData/etudes/STUD/2018/621853/EPRS_STU(2018)621853_EN.pdf
- https://ec.europa.eu/food/animals/pet-movement/eu-legislation/young-animals_en

Sweden:

- Directive 92/65/EEC (europa.eu) https://eur-lex.europa.eu/legal-content/DA/TXT/PDF/?uri=CELEX:01992L0065-20141229&from=EN
- Regulation (EU) 1/2005 (europa.eu) https://www.europarl.europa.eu/RegData/etudes/STUD/2018/621853/EPRS_STU(2018)621853_EN.pdf
- https://djur.jordbruksverket.se/swedishboardofagriculture/engelskasidor/trade/animals/dogscatsandferrets.4.73ec2c8614c1f08e28fbf273.html
- https://ec.europa.eu/food/animals/pet-movement/eu-legislation/young-animals_en

Norway:

- Forskrift om dyrehelsemessige vilkår for import og eksport av levende dyr, sæd, egg og embryo https://lovdata.no/dokument/SF/forskrift/2004-02-20-464
- Forskrift om ikke-kommersiell forflytning av kjæledyr https://lovdata.no/dokument/SF/forskrift/2016-05-19-542
- https://ec.europa.eu/food/animals/pet-movement/eu-legislation/young-animals_en
- Forskrift om import fra tredjestater av visse levende dyr, bier, humler og ferskt kjøtt av visse dyr https://lovdata.no/dokument/SF/forskrift/2010-07-23-1137
- Forskrift om hunder https://lovdata.no/dokument/SF/forskrift/2004-08-20-1204?q=forskrift%20om%20hund

England:

- Importing animals, animal products and high-risk food and feed not of animal origin from 1 January 2021 - GOV.UK (www.gov.uk)
- Commission Implementing Regulation (EU) 2019/2007 of 18 November 2019 laying down rules for the application of Regulation (EU) 2017/625 of the European Parliament and of the Council as regards the lists of animals, products of animal origin, germinal products, animal by-products and derived products and hay and straw subject to official controls at border control posts and amending Decision 2007/275/EC (Text with EEA relevance) (legislation.gov.uk)

- Bringing your pet dog, cat or ferret to the UK - GOV.UK (www.gov.uk)

The Netherlands:
- Directive 92/65/EEC (europa.eu) https://eur-lex.europa.eu/legal-content/DA/TXT/PDF/?uri=CELEX:01992L0065-20141229&from=EN
- Regulation (EU) 1/2005 (europa.eu) https://www.europarl.europa.eu/RegData/etudes/STUD/2018/621853/EPRS_STU(2018)621853_EN.pdf
- Wet dieren https://wetten.overheid.nl/BWBR0030250/2020-01-01/#Hoofdstuk2_Paragraaf1_Artikel2.2
- https://www.rvo.nl/onderwerpen/agrarisch-ondernemen/dieren/huisdieren-houden-en-fokken/honden-registreren-en-identificeren/honden-uit-het-buitenland
- https://ec.europa.eu/food/animals/pet-movement/eu-legislation/young-animals_en
- https://www.nvwa.nl/onderwerpen/honden-en-katten/regels-voor-stichtingen-die-honden-of-katten-uit-buitenland-invoeren

Germany:
- Directive 92/65/EEC (europa.eu) https://eur-lex.europa.eu/legal-content/DA/TXT/PDF/?uri=CELEX:01992L0065-20141229&from=EN
- Regulation (EU) 1/2005 (europa.eu) https://www.europarl.europa.eu/RegData/etudes/STUD/2018/621853/EPRS_STU(2018)621853_EN.pdf
- Gesetz zur Beschränkung des Verbringens oder der Einfuhr gefährlicher Hunde in das Inland https://www.gesetze-im-internet.de/hundverbreinfg/BJNR053010001.html
- https://ec.europa.eu/food/animals/pet-movement/eu-legislation/young-animals_en

Austria:
- Directive 92/65/EEC (europa.eu) https://eur-lex.europa.eu/legal-content/DA/TXT/PDF/?uri=CELEX:01992L0065-20141229&from=EN
- Regulation (EU) 1/2005 (europa.eu) https://www.europarl.europa.eu/RegData/etudes/STUD/2018/621853/EPRS_STU(2018)621853_EN.pdf
- https://ec.europa.eu/food/animals/pet-movement/eu-legislation/young-animals_en
- Tierkauf im Ausland | Europäisches Verbraucherzentrum Österreich (europakonsument.at)

Italy:
- Directive 92/65/EEC (europa.eu) https://eur-lex.europa.eu/legal-content/DA/TXT/PDF/?uri=CELEX:01992L0065-20141229&from=EN
- Regulation (EU) 1/2005 (europa.eu) https://www.europarl.europa.eu/RegData/etudes/STUD/2018/621853/EPRS_STU(2018)621853_EN.pdf
- https://ec.europa.eu/food/animals/pet-movement/eu-legislation/young-animals_en

The USA:
- Personal communication with Andrew Rowan, PhD, and Lisa Moses, VMD, DACVIM
- https://www.cdc.gov/importation/bringing-an-animal-into-the-united-states/dogs.html
- https://www.aphis.usda.gov/aphis/pet-travel/bring-pet-into-the-united-states/pet-travel-dogs-into-us
- https://www.aphis.usda.gov/animal_welfare/downloads/AC_BlueBook_AWA_508_comp_version.pdf

Australia:
- https://www.agriculture.gov.au/cats-dogs/step-by-step-guides

New Zealand:
- Dog control act https://legislation.govt.nz/act/public/1996/0013/latest/whole.html?search=ts_act%40bill%40regulation%40deemedreg_dog_resel_25_a&p=1#DLM374489
- https://www.mpi.govt.nz/bring-send-to-nz/pets-travelling-to-nz/bringing-cats-and-dogs-to-nz/step-by-step-guide-to-bringing-cats-and-dogs-to-nz/

9. Stray dogs

9.1 Table: Stray dogs

	Educational requirements for animal shelter staff	Requirements for the layout of animal shelters	Time limit for rehoming/killing
**Denmark**	The people responsible for animal shelters must have completed an education with focus on dog behaviour, care, legislation etc. The act states the subjects to be covered.<br><br>The Danish Veterinary and Food Administration approves the education.<br><br>An education may be replaced by documentation of appropriate experience within the area.	Requirements for commercial dog keeping, including animal shelters<br><br>● Prevention of disease<br>● Hygiene<br>● Records<br>● Layout of the premises<br>● Feeding and water<br>● Physical and mental stimulation<br>● Walking<br>● Human contact	3 days after search for owner has been initiated.
**Sweden**	Animal shelters must be considered as "a business" since there are typically more than 10 dogs.<br><br>Anyone engaged in commercial dog keeping should have adequate competencies acquired through education or similar experience.<br><br>The competencies must include the areas of dog behaviour, care, legislation etc.	No requirements specifically for commercial dog keeping.<br><br>Requirements for dog keeping in general:<br><br>● Meeting the dog's physical and mental needs<br>● Cage and room size<br>● Hygiene<br>● Feeding and water<br>● Layout of the premises<br>● Walking<br>● Human contact	5 days after the known owner has been informed.<br><br>10 days after Länsstyrelsen has been informed if the owner is unknown.
**Norway**	When selling and temporarily keeping animals, the person responsible for the company must be able to document the knowledge and skills necessary to manage animal welfare.	The following requirements apply to sale and temporary keeping of animals in general:<br><br>● Records<br>● Layout of the premises<br>● Prevention of disease<br>● Feeding and water<br>● Hygiene<br>● Consideration for the animal's behaviour, socialisation, health and age	Seven days after a search for owner has been initiated.

**England**	If animal shelters are considered as carrying out "sale, breeding, keeping", they are subject to the same requirements as breeders:<br>The person who has obtained the license as well as employees caring for the animals must have the competencies to identify normal behaviour and recognise, prevent and handle signs of pain, distress, injury, disease or unusual behaviour.	If animal shelters are considered as carrying out "sale, breeding, keeping", requirements apply to the following:<br>● The license must visible<br>● Records<br>● Layout of the premises<br>● Feeding and water<br>● Meeting the dog's physical and mental needs<br>● Training<br>● Protection against pain, distress, injury and disease	The police must contact the owner if it is identified, but the legislation does not state a time limit for rehoming.
**The Netherlands**	If animal shelters are considered as commercial animal keeping, they must obtain a license.<br>Diploma or certificate documenting an education with focus on the animal you work with.<br>The diploma or certificate must be issued from an accredited institution.	If animal shelters are considered as commercial animal keeping, requirements apply to the following:<br>● Records<br>● Layout of the premises<br>● Health<br>● Socialisation of young animals	2 weeks.
**Germany**	Breeding, keeping and selling animals in general are subject to non-specific requirements for acquiring and updating the knowledge required to keep, care for and euthanise animals.	No legislation identified.<br>Requirements for premises etc. in connection to dog keeping and breeding.	No legislation identified.
**Austria**	At least one person with a relevant specialist education is involved in managing the animal shelter.	Besides requirements for dog keeping, requirements for animals shelters with animals in general apply to the following:<br>● Cold and hot water in each facility<br>● Hygiene<br>● The layout must meet needs specific to the species<br>● Lighting and ventilation<br>● Sun protection<br>● Smoking should be forbidden<br>● If animals can move freely, it must be ensured that they do not harm themselves<br>● Veterinary inspection<br>● Prevention of disease<br>● Access to outdoor areas	1 month.

**Italy**	No legislation identified.	The regions establish criteria for the municipal animal shelters to ensure that they meet the requirements for hygiene and good living conditions for the dogs.	60 days.
**The USA**	Educational requirements in some states, but far from all states. There is a wish for a legal educational requirement for animal shelter staff.	No legislation identified.	Varies between states and municipalities, but typically 3-7 days.
**Australia**	<u>New South Wales</u><br>No legislation identified.<br><br>If the animal shelter can be considered as a "boarding establishment" (commercial housing of animals, including veterinary hospitals), it is required that the staff is experienced in handling the animals, and formal training/education is encouraged.	<u>New South Wales</u><br>General requirements for dog keeping in general:<br>● The animals must be offered feed, water and shelter<br>● The animals must be adequately exercised<br>● Animals cannot be tied up for an unreasonable period of time with an unreasonably heavy or short device<br><br>If the animal shelter can be considered as a "boarding establishment" (commercial housing of animals, including veterinary hospitals), more detailed requirements apply to the following:<br>● Layout<br>● Indoor climate (air, temperature, noise, light)<br>● Cage size<br>● Surface<br>● Hygiene<br>● Disease prevention, including an associated veterinarian<br>● Records<br>● Killing only via veterinarian<br>● Feed and water<br>● Exercise<br>● Transportation	<u>New South Wales</u><br>At least seven days.
**New Zealand**	No legislation identified.	Requirements for dog keeping in general, applicable to all who take care of or work with dogs:<br>● Feed and water<br>● Tying up (if applicable)<br>● A place to lie down<br>● Shelter and protection from heat and cold<br>● Hygiene	Varies between areas (councils), most commonly around seven days.

		In addition to the compulsory minimum requirements, best practice offers very specific guidelines.	

9.2 Links: Stray dogs

Denmark

- Bekendtgørelse af lov om hunde https://www.retsinformation.dk/eli/lta/2021/329
- Bekendtgørelse om dyrevelfærdsmæssige mindstekrav til hold af hunde https://www.retsinformation.dk/eli/lta/2020/1749

Sweden:

- Statens jordbruksverks föreskrifter och allmänna råd om hållande av hundar och katter https://www.hsb.se/contentassets/9b52bd940f684adab136bbc99f7b42f2/kalla-foreskrift-om-nya-regler-for-hundar-och-katter-sjvfs2020-8.pdf
- Föreskrifter om tillståndsplikt för viss djurhållning av sällskapsdjur, häst och pälsdjur http://djur.jordbruksverket.se/download/18.7c1e1fce169bee5214fb19d3/1554448722900/2019-027.pdf
- Lag om tillsyn över hundar och katter https://lagen.nu/2007:1150

Norway:

- Lov om dyrevelferd https://lovdata.no/dokument/NL/lov/2009-06-19-97
- Lov om hundehold https://lovdata.no/dokument/NL/lov/2003-07-04-74/KAPITTEL_3#KAPITTEL_3

England:

- The Animal Welfare (Licensing of Activities Involving Animals) (England) Regulations 2018 The Animal Welfare (Licensing of Activities Involving Animals) (England) Regulations 2018 (legislation.gov.uk)
- The Environmental Protection (Stray Dogs) Regulation 1992 https://www.legislation.gov.uk/uksi/1992/288/regulation/2/made

The Netherlands:

- Besluit houders van dieren https://wetten.overheid.nl/BWBR0035217/2018-07-01
- Burgerlijk Wetboek Boek 5 https://wetten.overheid.nl/BWBR0005288/2018-09-19

Germany

- Tierschutzgesetz http://www.gesetze-im-internet.de/tierschg/BJNR012770972.html
- Tierschutz-Hundeverordnung http://www.gesetze-im-internet.de/tierschhuv/index.html#BJNR083800001BJNE000100305

Austria:

- Bundesrecht konsolidiert: Gesamte Rechtsvorschrift für Tierschutzgesetz https://www.ris.bka.gv.at/GeltendeFassung.wxe?Abfrage=Bundesnormen&Gesetzesnummer=20003541
- Bundesrecht konsolidiert: Gesamte Rechtsvorschrift für 2. Tierhaltungsverordnung https://www.ris.bka.gv.at/GeltendeFassung.wxe?Abfrage=Bundesnormen&Gesetzesnummer=20003860
- Bundesrecht konsolidiert: Gesamte Rechtsvorschrift für Tierschutz-Sonderhaltungsverordnung https://www.ris.bka.gv.at/GeltendeFassung.wxe?Abfrage=Bundesnormen&Gesetzesnummer=20010231

Italy:

- Legge 281/1991 http://www.salute.gov.it/imgs/C_17_normativa_911_allegato.pdf

The USA:
- https://www.animallaw.info/article/frequently-asked-questions-local-dog-laws
- https://www.animallaw.info/article/detailed-discussion-state-dog-impound-laws#id-5

Australia:
- https://legislation.nsw.gov.au/view/whole/html/inforce/current/act-1979-200#sec.8
- https://www.dpi.nsw.gov.au/animals-and-livestock/animal-welfare/general/welfare-of-dogs/aw-code-5

New Zealand
- Code of welfare – dogs https://www.mpi.govt.nz/dmsdocument/1428-Dogs-Animal-Welfare-Codes-of-Welfare
- https://www.aucklandcouncil.govt.nz/dogs-animals/lost-adoptable-pets/Pages/get-impounded-dog-back.aspx
- https://www.napier.govt.nz/services/animal-education/napier-dog-pound/
- https://www.ashburtondc.govt.nz/services/dogs-and-animals/dogs-lost-and-found
